# 4-phenylbutyric acid alleviated sepsis-induced myocardial dysfunction by inhibition of MAM formation to improve mitochondrial dynamic balance in myocardial cells

**DOI:** 10.1093/burnst/tkag039

**Published:** 2026-06-03

**Authors:** Yue Wu, Yijin Fang, Yuanqun Zhou, Qinghui Li, Liyong Zou, Xiaodan Wang, Li Wang, Ying Zheng, Liangming Liu, Yu Zhu

**Affiliations:** State Key Laboratory of Trauma and Chemical Poisoning, Department of Shock and Transfusion, Daping Hospital, Army Medical University, No. 10 Changjiang Branch Road, Yuzhong District, Chongqing 400042, China; Department of Gynecologic Oncology, West China Second University Hospital, Sichuan University, No. 141 Renmin South Road Section 3, Wuhou District, Chengdu City, Sichuan Province 610041, China; State Key Laboratory of Trauma and Chemical Poisoning, Department of Shock and Transfusion, Daping Hospital, Army Medical University, No. 10 Changjiang Branch Road, Yuzhong District, Chongqing 400042, China; State Key Laboratory of Trauma and Chemical Poisoning, Department of Shock and Transfusion, Daping Hospital, Army Medical University, No. 10 Changjiang Branch Road, Yuzhong District, Chongqing 400042, China; State Key Laboratory of Trauma and Chemical Poisoning, Department of Shock and Transfusion, Daping Hospital, Army Medical University, No. 10 Changjiang Branch Road, Yuzhong District, Chongqing 400042, China; State Key Laboratory of Trauma and Chemical Poisoning, Department of Shock and Transfusion, Daping Hospital, Army Medical University, No. 10 Changjiang Branch Road, Yuzhong District, Chongqing 400042, China; State Key Laboratory of Trauma and Chemical Poisoning, Department of Shock and Transfusion, Daping Hospital, Army Medical University, No. 10 Changjiang Branch Road, Yuzhong District, Chongqing 400042, China; Department of Gynecologic Oncology, West China Second University Hospital, Sichuan University, No. 141 Renmin South Road Section 3, Wuhou District, Chengdu City, Sichuan Province 610041, China; State Key Laboratory of Trauma and Chemical Poisoning, Department of Shock and Transfusion, Daping Hospital, Army Medical University, No. 10 Changjiang Branch Road, Yuzhong District, Chongqing 400042, China; State Key Laboratory of Trauma and Chemical Poisoning, Department of Shock and Transfusion, Daping Hospital, Army Medical University, No. 10 Changjiang Branch Road, Yuzhong District, Chongqing 400042, China

**Keywords:** Sepsis, 4-phenylbutyric acid, Myocardial dysfunction, Mitochondrial dynamics, MAM, Lactylation, Hexokinase 2, Endoplasmic reticulum stress

## Abstract

**Background:**

Sepsis-related myocardial dysfunction significantly increases the mortality risk of sepsis. However, its underlying mechanism remains incompletely understood, and effective therapeutic strategies are still lacking. Transcriptomic profiling from septic patients showed endoplasmic reticulum stress (ERS) were the main pathways participating in the occurrence of sepsis myocardial dysfunction. Therefore, this study aimed to explore the protective effect of 4-phenylbutyric acid (4-PBA) on sepsis-induced myocardial injury and specify its molecular regulatory mechanism, to provide experimental basis for clinical intervention of septic myocardial dysfunction.

**Methods:**

*In vivo* and *in vitro* models of sepsis were used, and 4-PBA (5 mg/kg) was administered for intervention. Mitochondria-associated ER-membrane (MAM) formation, mitochondrial dynamics, mitochondrial function, glycolytic metabolism, and protein lactylation were systematically examined. Molecular docking and site-directed mutation were applied to verify the direct binding and catalytic sites of 4-PBA.

**Results:**

4-PBA significantly alleviated sepsis-induced myocardial dysfunction (SIMD). The mechanism was closely related to 4-PBA inhibiting MAM formation and improving the mitochondrial dynamic balance and mitochondrial function. 4-PBA inhibited MAM formation mainly via decreasing the lactate production and lactylation of Arpc1b-K308 by inhibiting the glycolysis limiting enzyme HK2, and 4-PBA inhibited HK2 activity by binding K621 and K624 catalytic sites.

**Conclusions:**

The results indicate that 4-PBA protects cardiac function following sepsis by recovering mitochondrial dynamics balance. This finding provides a novel therapeutic strategy and potential target for SIMD.

## Graphical Abstract

**Graphical Abstract:**
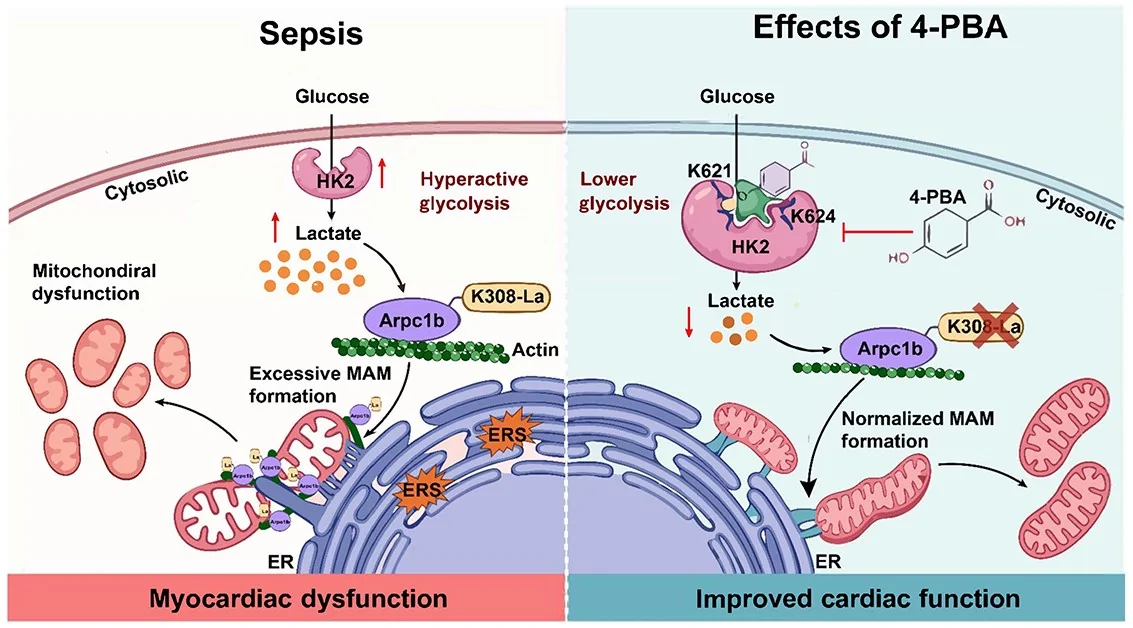

## Highlights

- 4-PBA markedly improves sepsis-induced myocardial dysfunction via restraining excessive MAM formation and restoring myocardial mitochondrial dynamics homeostasis.
- 4-PBA directly binds the K621 and K624 catalytic residues of HK2 to suppress HK2 enzymatic activity, resulting in inhibited glycolysis and decreased intracellular lactate accumulation in cardiomyocytes.
- HK2 inhibition-mediated lactate reduction lessens Arpc1b K308 lactylation, which subsequently abrogates aberrant MAM assembly in septic cardiomyocytes.
- This study identifies a previously unrecognized regulatory cascade: HK2-lactylated Arpc1b-MAM-mitochondria signaling, which provides a potential therapeutic strategy for sepsis-induced myocardial injury.

## Background

Sepsis-induced myocardial dysfunction (SIMD) is a frequent and serious complication of the condition. Studies have shown that ~40% of patients develop cardiac dysfunction after sepsis, manifested as the impairment of myocardial contractility, heart failure, and other symptoms [1]. SIMD not only serves as a major cause of multiple organ failure but also significantly increases the mortality risk of sepsis patients. The reports showed that the mortality rate was ~70%–90% in sepsis patients with cardiovascular dysfunction [2]. Although several strategies were proposed, such as the application of inflammatory mediator scavengers and myocardial calcium sensitizers [3], the outcomes remained unsatisfactory. Previous studies showed that endoplasmic reticulum stress (ERS) participated in the pathogenesis of a variety of diseases, such as cancer, inflammatory diseases, metabolic diseases, and neurodegenerative diseases [4, 5]. At the same time, inhibitions of ERS via taurine and 4-phenylbutyric acid (4-PBA) were shown to be beneficial to diseases, including Alzheimer’s disease, diabetes, myocardial ischemia–reperfusion injury and so on [6–8]. Whether inhibition of ERS is beneficial to SIMD and its mechanisms are unclear.

Basic researches showed that myocardial contractility functions relied on energy supply and the myocardial proteins. The mitochondria are powerhouse of cell, and mitochondria in myocardial cell account for 40% of the total cell volume in cardiomyocyte which produce 6 kg of adenosine triphosphate (ATP) daily for a person [9]. Number of studies demonstrate that mitochondrial dynamics is the decisive factor for mitochondrial function, and our previous studies showed that sepsis led to mitochondrial dynamics imbalance which presented mitochondrial fragments and dysfunction. And researched testified that the endoplasmic reticulum encircling mitochondria initiated mitochondrial fission and regulated mitochondrial dynamics balance. However, it is not clear whether protective effects of ERS inhibition are related with mitochondrial dynamics balance.

In present study, with cecum ligation and puncture (CLP) induced sepsis models in mice and rats, sepsis serum-incubation of cardiomyocytes model, multi-omics analysis including metabolomics, proteomics, and modification omics, the protective effects of the ERS inhibition on septic cardiac function were investigated, and the mechanisms involving in the formation of endoplasmic reticulum–mitochondria membrane contract sites, mitochondrial dynamics, and mitochondrial function were further explored.

## Methods

### Ethic approval

All animal protocols complied with the Animal Research: Reporting of *In vivo* Experiments guidelines and were approved by the Institutional Animal Care and Use Committee of Army Medical University (Approval No. AMUWEC20223185).

### Reagents

Antibodies for Mfn2 (ab56889), Drp1 (ab184247), OPA1 (ab42364), and GAPDH (ab8245) were acquired from Abcam (Cambridge, MA, USA). The primary antibody for β-actin (BM0627) was purchased from Boster (Wu Han, China). Antibodies for annexin was purchased from BD Pharmingen (California, USA). Antibodies for lactyl-Arpc1b (Lys308) (CL121501) was obtained from PTM Biolabs (Hangzhou, China). Alexa Fluor 790-Affinipure goat anti-rabbit (111-655-144) and Alexa Fluor 790-Affinipure goat anti-mouse (115-655-146) were obtained from Jackson (Pennsylvania, USA). MitoTracker™ Deep Red (M22426) and MitoTracker™ Deep Green (M7514) were obtained from Thermo Fisher (Waltham, MA, USA). DCFH-DA (S0033M), JC-1 (C2003S), and ATP (S0027) assay kits were acquired from Beyotime Biotechnology (Shanghai, China). Lactate assay kit (bc2235) was acquired from Solarbio (Beijing, China). TNFα, IL 1β, and bicinchoninic acid (BCA) assay kit were acquired from Beyotime Biotechnology (Shanghai, China). Mitochondrial deoxyribonucleic acid (MtDNA; KB12100) assay kit were purchased from Kehbio (Beijing, China). 4-PBA, BSA, taurine, and sodium pentobarbital were obtained from Sigma-Aldrich (St. Louis, Missouri, USA). Salubrinal was purchased from Cayman (USA). TUDCA was purchased from Solarbio (Beijing, China). Reagents related to cellular energy metabolism, such as oligomycin, FCCP, antimycin A, and rotenone were purchased from Agilent (USA). Advanced DNA Transfection Reagent was purchased from Zeta life (CA, USA). siRNA for Arpc1b-K308 (K to T), Anxa6-K299 (K to T), Cald1-K524 (K to T), Cttn-K255 (K to T), Septin5-K10 (K to T), and Septin9-K154 (K to T) were generated by RiboBio (Guangzhou, China). Lentiviral vectors for Arp2/3 K308 mutations (K308T and K308R) were generated by Obio life Technology (Shanghai, China). Primers for ND1, ND6, COX1, and D-LOOP were purchased from Invitrogen (CA, USA). Blaze TaqTM SYBR Green quantitative polymerase chain reaction (qPCR) mix2.0 was obtained from GeneCopoeia (Rockville, Maryland, USA). Unless otherwise stated, all additional chemicals were obtained from Sigma-Aldrich.

### Dataset collection and analysis

#### Dataset collection

Gene expression profiles used in this study were retrieved from the publicly available Gene Expression Omnibus (GEO) database (https://www.ncbi.nlm.nih.gov/geo/). Two independent datasets were selected for downstream analysis: GSE65682 and GSE79962. The GSE65682 dataset included 760 patients with sepsis and 42 healthy controls, while GSE79962 consisted of 20 septic patients and 11 healthy individuals. Both datasets were utilized to conduct differential gene expression analysis.

#### Differentially expressed genes analysis

Transcriptomic datasets GSE65682 and GSE79962 were downloaded from the publicly available GEO database (https://www.ncbi.nlm.nih.gov/geo/). Batch effect correction was performed using the Combat function from the SVA package. Differentially expressed genes (DEGs) were detected using the limma analysis, applying the cut-offs of |fold change (FC)| > 1.5 and *P*-value <.05.

#### Weighted gene co-expression network analysis

Weighted gene co-expression network analysis (WGCNA) was constructed by the R package “WGCNA” to identify the co-expression modules most strongly associated with sepsis patients. Briefly, the dataset was first preprocessed; subsequently, a correlation matrix was generated using the WGCNA package. We set the soft-threshold power to 5 to create the adjacency matrix, which was further applied to develop the topological overlap matrix. Average linkage hierarchical clustering was employed to identify gene modules on similar expression patterns. Genes from the module most strongly associated with sepsis were selected for functional enrichment analysis.

#### Myocardial contraction score and endoplasmic reticulum stress score

Myocardial contraction score and ERS score was calculated using the AddModuleScore function in the Seurat R package. Specifically, based on the previous single-cell transcriptome data of mouse myocardial tissue, the cardiomyocyte expression matrix was extracted and further subjected to dimensionality reduction and clustering using the “Seurat” package to obtain cardiomyocyte subpopulations. A cardiomyocyte contraction-related gene set (230 genes) and an ERS-related gene set (730 genes) were constructed by collecting relevant genes from published reports and the MSigDB repository (https://www.gsea-msigdb.org/gsea/msigdb/genesets.jsp), respectively. The AddModuleScore function was utilized for the quantification of the gene sets expression levels in each cardiomyocyte, thereby assessing the contraction capacity and ERS level of cardiomyocytes—with higher scores indicating stronger contraction function or more severe ERS. The Wilcoxon rank-sum test was employed to assess intergroup differences, with results presented as box plots.

### Animal and cell model preparation

#### Animal preparation

C57BL/6 and Slc16a1-creEsr1 were purchased from Cyagen Biosciences Inc. (Suzhou, China). Adult Sprague–Dawley rats of both sexes (12 weeks old, weighing 220 ± 10 g) were obtained from the Laboratory Animal Center of the Daping Hospital (Army Medical Center). Animals used in this study were maintained under specific pathogen-free housing conditions at the Laboratory Animal Center of Army Medical Center. The environment was strictly controlled at a temperature of 22 ± 2°C, relative humidity of 50 ± 5%, and a 12 h light/12 h dark cycle. Standard laboratory chow and water were provided ad libitum. Before experiments, all mice were acclimated to the environment for 1 week.

### Sepsis model establishment and septic serum preparation

Animals were anesthetized via intraperitoneal administration of sodium pentobarbital at a dose of 45 mg/kg. The sepsis model was established using the cecal ligation and puncture (CLP) procedure, as detailed in our previous work [10]. Briefly, a midline laparotomy was performed to expose the cecum, which was then ligated 0.5 cm proximal to its distal tip, followed by puncture at the distal tip: a triangular needle was used for rats, while a 20-gage needle was used for double punctures in mice. Feces were allowed to enter the peritoneal space. Subsequently, the abdomen was sutured and the animals were returned to their cages with free access to water but no food. For the sham-operated group (Sham), only the abdominal incision and closure were performed without CLP. Twelve hours after CLP, the animals with mean arterial pressure (MAP) of <70 mmHg or a > 30% reduction in MAP compared with preoperative values were defined as model establishment successful and were used for subsequent experiments. The model establishment induction rate was 85% in present study.

### Cell culture and cell model of sepsis rat serum stimulation

The rat heart-derived cell line clone 9C2 (H9C2) was purchased from the American Type Culture Collection (ATCC). The H9C2 cells were maintained in high-glucose medium containing 10% fetal bovine serum (FBS) and incubated at 37°C in a 5% CO₂ humidified atmosphere. During experiments, H9C2 cells were maintained in serum-free low-glucose Dulbecco’s Modified Eagle Medium (DMEM; containing 1000 mg/L D-glucose) and stimulated with 10% (v/v) sterile sepsis rat serum (collected from CLP-induced septic rats with successful model establishment). After 24 h stimulation, 5 mM 4-PBA was supplemented and incubated for 4 h in the 4-PBA group, the control group used same amount of solvent of 4-PBA.

### Single-cell nucleus ribonucleic acid sequencing analysis

Three mice from each group (sham-operated group and sepsis group). Under sodium pentobarbital anesthesia (45 mg/kg, intraperitoneal injection), a thoracotomy was performed and the heart was rapidly perfused with ice-cold (0°C) PBS until complete blood clearance. The left ventricle was excised, rinsed in pre-chilled phosphate buffered saline with EDTA (PBSE), minced and transferred to GEXSCOPE® Nuclear Isolation Solution for nuclear isolation based on the manufacturer’s instructions. The isolated nuclear pellet was resuspended in PBSE, filtered through a 40 μm strainer to obtain a single-nucleus suspension, and quantified by Trypan blue exclusion. Nuclei were stained with DAPI (1:1000) to test the integrity.

A single-nucleus suspension with a density 3–4 × 10^5^ nuclei/ml was added to the microfluidic chip. Amplification of cDNA and library preparation were conducted with the Singleron single-nucleus RNA-sequencing kit (snRNA-seq). The Illumina NovaSeq 6000 system was applied for library sequencing, and CeleScope v1.9.0 was used to process raw reads for filtering, alignment, and quantification to generate the gene-expression matrix. Data were loaded into the Seurat package (R 4.3.2) for dimensionality reduction, clustering, and differential analysis. Nuclei with UMI > 30 000, gene counts <200 or > 5000, or mitochondrial content >50% were excluded. Normalization and standardization of gene expression data were conducted via NormalizeData and ScaleData. The top 2000 highly variable genes were screened using FindVariableFeatures for principal component analysis (PCA), and the first 15 principal components were adopted for cell clustering. Batch effects were mitigated with Harmony, followed by unsupervised clustering (FindClusters) and visualization by t-SNE. Marker genes for each cluster were identified (FindMarkers), and cell-type annotation was performed by cross-referencing the SynEcoSys database. Cardiomyocyte nuclei were subsetted for subsequent sub-clustering analyses.

### Cardiac output detection of rats

Cardiac output (CO) in rats was measured using thermodilution technique. Specifically, a thermodilution catheter was positioned in the left ventricle by way of the right common carotid artery in rats, while another catheter was placed in the jugular vein to facilitate normal saline injection. The injection temperature and volume of normal saline are critical factors affecting CO measurement: the temperature of normal saline was automatically detected by a temperature probe, and the injection volume was set at 0.3 ml. During the experiment, the catheters were connected to a CO analyzer (Cardio MAX, Model 0162-004 M), which was used to determine CO.

### Echocardiography detection of mice and rats

Animals were anesthetized with 1% isoflurane inhalation, and transthoracic echocardiography was carried out on a Vivid 9 high-frequency color Doppler device. Assessment of left ventricular systolic function was performed via the parasternal long-axis view, with measurements acquired using M-mode echocardiography. Left ventricular functional parameters, namely left ventricular fractional shortening (LVFS) and left ventricular ejection fraction (LVEF), were calculated.

### Mitochondrial morphology observation by transmission electron microscopy

Fresh left-ventricular myocardium was harvested for ultrastructural analysis of mitochondrial morphology. In total, 1 mm^3^ tissue blocks were rapidly fixed in 2.5% glutaraldehyde buffered with 0.1 M phosphate (pH 7.4) at 4°C for 4 h, rinsed three times in 0.2 M PBS, and post-fixed in 1% osmium tetroxide for 2 h at 4°C. After graded ethanol dehydration (50%, 70%, 90%, 100% × 3, 10 min each) and propylene oxide infiltration, Epoxy resin was used for tissue embedding. After staining with uranyl acetate and lead citrate, the ultrathin sections were visualized with a Hitachi H-7500 transmission electron microscope. Mitochondrial density (mitochondrial area per unit cytosolic area) and the mitochondrial aspect ratio (major/minor axis length) were calculated using Image J.

### Mitochondrial morphology observation by confocal microscopy

H9C2 cells were cultured on 20-mm glass-bottom confocal dishes until they reached 80% confluence. MitoTracker™ Deep Red FM was dissolved in high-purity DMSO to a 1 mM stock, aliquoted, and stored at −20°C away from light. The stock solution was diluted 1:10000 (final 100 nM) in serum-free, high-glucose DMEM. Cells were gently rinsed three times with preheated 37°C PBS, following a 15-min incubation with 1 ml staining solution at 37°C and 5% CO₂, additional three washes with sterile PBS were performed. An inverted confocal microscope with a 63× oil objective was used to observe mitochondrial structure. MitoTracker™ Deep Red FM was excited at 633 nm, and the resulting fluorescence was captured in the range of 655 to 670 nm.

### Time-lapse monitoring of mitochondrial dynamics in live cells

Cells were cultured as previously reported. When they reached 80% confluence. Cells were treated with MitoTracker™ Deep Red (1:10 000) and incubated for 20 min in a 5% CO₂ incubator at 37°C. After incubation and three PBS washes, the medium was replaced with high-glucose DMEM without serum. We performed time-lapse observation using a confocal laser scanning microscope. Images were captured at 30 s intervals for a total duration of 10 min. The continuous images were analyzed to count the total number of mitochondrial fission and fusion events within the same field of view over the 10 min period. Mitochondrial branched length and mitochondrial fission or fusion frequency were quantified using ImageJ.

### Mitochondria-associated endoplasmic reticulum–membrane measurements

Cardiomyocytes (H9C2) were cultured on 20-mm glass-bottom confocal dishes until they reached 80% confluence. To observe ER, cells were incubated with ER-Tracker™ Green (1:1000 in pre-warmed Hank’s balanced salt solution) for 30 min in a 5% CO₂ humidified incubator maintained at 37°C. Mitochondria were subsequently counterstained with MitoTracker™ Deep Red FM (100 nM) for a further 30 min under identical conditions. After three gentle washes with warm PBS, the medium was exchanged for phenol-red-free, serum-free DMEM to minimize background fluorescence. The formation of MAM was observed using excitation wavelengths of 488 nm and 633 nm on a confocal microscope. The proportion of the formation of MAM relative to the total cell area was quantified using ImageJ software.

### Measurement for mitochondrial oxygen consumption rate of cardiomyocytes (H9C2)

Oxygen consumption rate (OCR) was detected using H9C2 cardiomyocytes. Cells were plated in 24-well XFe microplates at a density of 2 × 10^4^ cells/well. Following seeding, the microplate was left on a laminar flow hood for 1 h to allow the cells to settle spontaneously. It was incubated overnight to promote firm cell attachment. With a cell confluence of 70%, the H9C2 cells were treated with 4-PBA and sepsis rat serum. Then the cells were incubated for 1 h in a basal medium supplemented with 2.5 μM glucose and 2 mM glutamine. Thereafter, cells were sequentially treated with medium supplemented with 2 μM oligomycin, 1 μM FCCP, and 0.5 μM rotenone/antimycin A. The OCR of H9C2 cells was then accurately measured using an extracellular flux analyzer.

### Mitochondrial function assessment

In total, 20 mm confocal dishes were used for culturing H9C2 cells. After incubation with JC-1 (1:200, 37°C for 30 min) or reactive oxygen species (ROS) indicator (100 μM, 37°C for 10 min), mitochondrial function was visualized via Leica TCS SP5 laser confocal microscopy. JC-1 monomers emitted green fluorescence (488 nm excitation, 501–563 nm emission), and aggregates produced red fluorescence (633 nm excitation, 655–670 nm emission). The red/green fluorescence ratio was applied to assess mitochondrial membrane potential. ROS fluorescence was captured at 800 V with 488 nm excitation and 501–563 nm emission. ImageJ was adopted to quantify mitochondrial membrane potential and ROS levels.

### Lentiviral construction and cell transfections

To overexpress the Arpc1b (K308T) and Arpc1b (K308R) mutants, a lentiviral packaging system was utilized. The recombinant vectors pSLenti-SFH-P2A-Puro-CMV-Arpc1b (K308T)-WPRE and pSLenti-SFH-P2A-Puro-CMV-Arpc1b (K308R)-WPRE were transfected into H9C2 cells following the manufacturer’s protocols, for the establishment of cell lines stably overexpressing the Arpc1b mutants. Transfected cells were selected with 2 μg/ml puromycin and subsequently cultured in maintenance medium supplemented with 1 μg/ml puromycin.

### Adeno-associated virus serotype 9 vector construction and *in vivo* transfection

Recombinant adeno-associated virus serotype 9 (AAV9) vectors containing the cardiac-specific cTNT promoter driving wild-type Arpc1b (AAV9-cTNT-mArpc1b-WT), lactylation-deficient mutant Arpc1b-K308R (AAV9-cTNT-mArpc1b(K308R)-3Flag-P2A-EGFP), lactylation-deficient mutant Arpc1b-K308T (AAV9-cTNT-mArpc1b(K308T)-3Flag-P2A-EGFP) and empty vector (AAV9-cTNT-mArpc1b-3Flag-P2A-EGFP) were constructed by Obio Technology (Shanghai, China). The virus titer was 1 × 10^12^ vg/ml. C57BL/6 mice (8-week-old) were injected with 100 μL of AAV9 vector via tail vein. Four weeks after transfection, the cardiac transduction efficiency was verified by immunofluorescence staining of EGFP. Sepsis models were established by CLP surgery, and cardiac function and mitochondrial function were detected 12 h after surgery.

### Analysis of liquid chromatography–tandem mass spectrometry metabolomics

Following treatment with septic rat serum and 4-PBA, 1 × 10^7^ H9C2 cells were centrifuged to form pellets and dried naturally. We added 200 μL deionized water to the pellets and froze them instantly in liquid nitrogen for 1 min. After thawing, the mixture was shaken vigorously for 30 s and disrupted by ultrasound in an ice bath for 10 min. We used 50 μL of the lysate for BCA protein assay, and transferred the rest into a 2 ml EP tube. After adding methanol and acetonitrile (300 μL each), the mixture was mixed well and ultrasonicated at 0°C for 15 min. Samples were stored at −40°C for 1 h before centrifugation at 12 000 rpm and 4°C for 15 min. The final supernatant was used for subsequent liquid chromatography-mass spectrometry (LC–MS) detection. Quality control (QC) samples were prepared by mixing supernatants from all groups and treated in the same way. All specimens were analyzed via the AB SCIEX UPLC-Triple TOF platform. Progenesis QI was used to process original data, and the exported comma-separated values (CSV) matrix was used for variance analysis. PCA and orthogonal partial least squares discriminant analysis were established using ropls 1.6.2 in R language, and 7-fold cross-validation was used to assess model performance. We also carried out Student’s *t-*test and FC calculation. Metabolites satisfying VIP > 1 and *P* < .05 were defined as significantly differential molecules. Database mining, functional enrichment and pathway annotation were then performed to explore the biological pathways linked to these metabolites.

### Analysis of liquid chromatography–tandem mass spectrometry proteomics

H9C2 cells were harvested to conduct proteomic profiling. We first formulated cell lysis solution consisting of 8 M urea, 1% protease inhibitor cocktail, 3 μM TSA and 50 mM NAM. This lysis buffer was mixed with cell samples at a volume ratio of 4:1, followed by ultrasonic treatment to fully disrupt cells. The resulting lysate was spun down at 12 000 g and 4°C for 10 min, and the supernatant was retained for subsequent experiments. Protein content in the supernatant was quantified with a commercial BCA assay kit. We extracted equivalent total protein from each sample and standardized the final volume using the prepared lysis buffer. Trichloroacetic acid was supplemented to reach a final concentration of 20%, and the mixture was kept at 4°C for 2 h to induce protein precipitation. The precipitated protein pellets were recovered via centrifugation at 4500 g for 5 min, and the upper liquid was removed completely. The pellets were rinsed two to three times with pre-cooled acetone and air-dried afterwards, then redissolved in 200 mM TEAB solution. Trypsin was added at an enzyme-to-protein mass ratio of 1:50, and the system was incubated overnight to complete protein digestion. Next, dithiothreitol (DTT) was applied to break disulfide bonds, with the reaction maintained at 56°C for 30 min. Iodoacetamide was then introduced, and samples were placed at room temperature away from light for 15 min to achieve alkylation of cysteine residues.

The obtained peptides were reconstituted with mobile phase A and separated on a NanoElute liquid chromatography system. Eluted peptides were ionized and further analyzed using a timsTOF Pro2 mass spectrometer. The secondary mass spectra were recorded within the mass-to-charge (m/z) range of 100 to 1700. Data collection adopted the parallel accumulation serial fragmentation (PASEF) mode. Each primary mass spectrum was analyzed through 10 cycles under PASEF settings, and secondary signals were captured for precursor ions carrying 0 to 5 charges. The dynamic exclusion duration for tandem mass detection was set to 30 s. All raw mass spectral files were imported into MaxQuant (version 1.6.15.0) for database matching and protein identification. PCA was performed to evaluate the overall quality and reproducibility of protein expression data. We further screened differentially expressed proteins (DEPs) and carried out functional enrichment analysis to explore the biological roles of these molecules.

### Molecular docking of 4-phenylbutyric acid and hexokinase 2

The 3D structure of hexokinase 2 (HK2) was downloaded from AlphaFold database. Meanwhile, the structural file of 4-PBA in SDF format was downloaded from PubChem. Both HK2 and 4-PBA files were converted into PDBQT format and loaded into PyMOL. We defined a grid box to cover the potential ligand binding site of the protein, then ran molecular docking simulation. The binding patterns were displayed via PyMOL to generate 3D interaction graphs. Additionally, LigPlot+ was applied to analyze the docking outcomes and plot the two-dimensional interaction profile between 4-PBA and HK2.

### Molecular dynamics simulation of lactoyl coenzyme A (lactyl-CoA) and actin-related protein 2/3 complex subunit 1B (Arpc1b)

Molecular docking simulation was carried out to characterize the binding interaction between lactyl-CoA and Arpc1b (UniProt ID: O88656), and the results confirmed that the lactyl group could bind to the 308th amino acid site of Arpc1b. Prior to formal simulation analysis, the whole complex system was structurally optimized via the conjugate gradient algorithm for energy minimization. To stabilize the simulated system and reach equilibrium state, two classical ensemble modes were applied sequentially, including the canonical ensemble (NVT, constant temperature and volume) and isobaric-isothermal ensemble (NPT, constant temperature and pressure). The particle mesh Ewald (PME) approach was utilized for accurate calculation of electrostatic interactions within the system, followed by a 100-nanosecond molecular dynamics (MD) simulation. Upon completion of the simulation, the GROMACS “gmx rms” command was adopted to analyze and extract the root-mean-square deviation (RMSD) and root-mean-square fluctuation (RMSF) values of the lactyl group and Arpc1b protein throughout the simulation process. Furthermore, we statistically analyzed the number of hydrogen bonds formed between the lactylation modification locus and its adjacent functional groups, and quantified the binding interaction energy of the complex simultaneously.

### Detection of lactylation proteomics

Total proteins extracted from H9C2 cells were processed via trypsin hydrolysis, coupled with reduction and alkylation treatments, to obtain mixed peptide fragments. The resulting peptide samples were resuspended in immunoprecipitation (IP) buffer (50 mM Tris–HCl, 100 mM NaCl, 1 mM EDTA, 0.5% NP-40, pH 8.0) and incubated with pre-rinsed PTM-1404 lactylation enrichment resin (PTM Bio, Hangzhou). The reaction system was placed on a rotary shaker and incubated overnight at 4°C to fully capture lactylated peptides. After the incubation procedure, the resin was rinsed four times with IP buffer and subsequently washed twice with deionized water to remove non-specifically bound impurities. Target peptides were eluted repeatedly three times using 0.1% trifluoroacetic acid eluent. All eluted fractions were pooled and concentrated by vacuum drying. The dried peptide samples were further desalted with C18 ZipTips following standard manufacturer protocols and subjected to secondary vacuum drying for subsequent detection. Purified peptides were redissolved in mobile phase A (2% acetonitrile containing 0.1% formic acid) and fractionated using the NanoElute ultra-high-performance liquid chromatography system. A linear gradient of mobile phase B (pure acetonitrile supplemented with 0.1% formic acid) was adopted for peptide elution at a constant flow rate of 450 nL/min. Separated peptides were ionized via a capillary ion source with a voltage of 1.6 kV and delivered into the timsTOF Pro mass spectrometer for qualitative and quantitative analysis.

The mass spectrometer was operated under the PASEF mode. For each full-scan primary mass spectrum, 10 secondary fragment spectra were collected targeting precursor ions with charge states ranging from 0 to 5. The mass scanning range was set from 100 to 1700 m/z, and the dynamic exclusion duration for tandem scanning was configured as 30 s to improve detection accuracy and repeatability.

### Statistical analysis

All quantitative data in this study are presented as mean values with standard deviation (mean ± SD). Statistical analysis was implemented with SPSS 20.0 software. Independent samples *t-*test was applied for statistical comparison between two groups. For datasets involving three or more experimental groups, one-way analysis of variance was adopted, and Tukey’s multiple comparison test was used for subsequent pairwise post-hoc analysis. In this work, a *P*-value <.05 was defined as the threshold for statistically significant differences.

## Results

### The relationship of endoplasmic reticulum stress with septic myocardial dysfunction

To evaluate the relationship of ERS with sepsis induced cardiac damage, the transcriptomic dataset in peripheral blood from sepsis patients (GSE65682) was obtained in the GEO public database, including 802 patients (42 healthy persons and 760 sepsis patients, Figure S1a). Sample distribution by PCA is shown in Figure S1b. With |FC| ≥ 1.5 and *P* < .05 as screening criteria, the 2743 differential-expressed genes (DEGs) were identified, including 1168 up-regulated and 1575 down-regulated in sepsis group (Figure S1c). A gene network was constructed via WGCNA and according to the scale-free network structure, the optimal threshold was selected as 5 (Figure S1d and e), and defining 10 modules (Figure S1f). Correlation analysis between modules and traits revealed that the red module genes were highly positively correlated with sepsis (Cor = 0.63, *P* = 7.9e-88) (Figure S1g and h, Cor = 0.77, *P* < 1e-200). Extracting 783 genes from the red module for functional enrichment analysis showed there were significant enrichment in ERS, glycolytic, and nucleotide metabolism pathway in sepsis patient group (Figure S1i).

**Figure 1:**
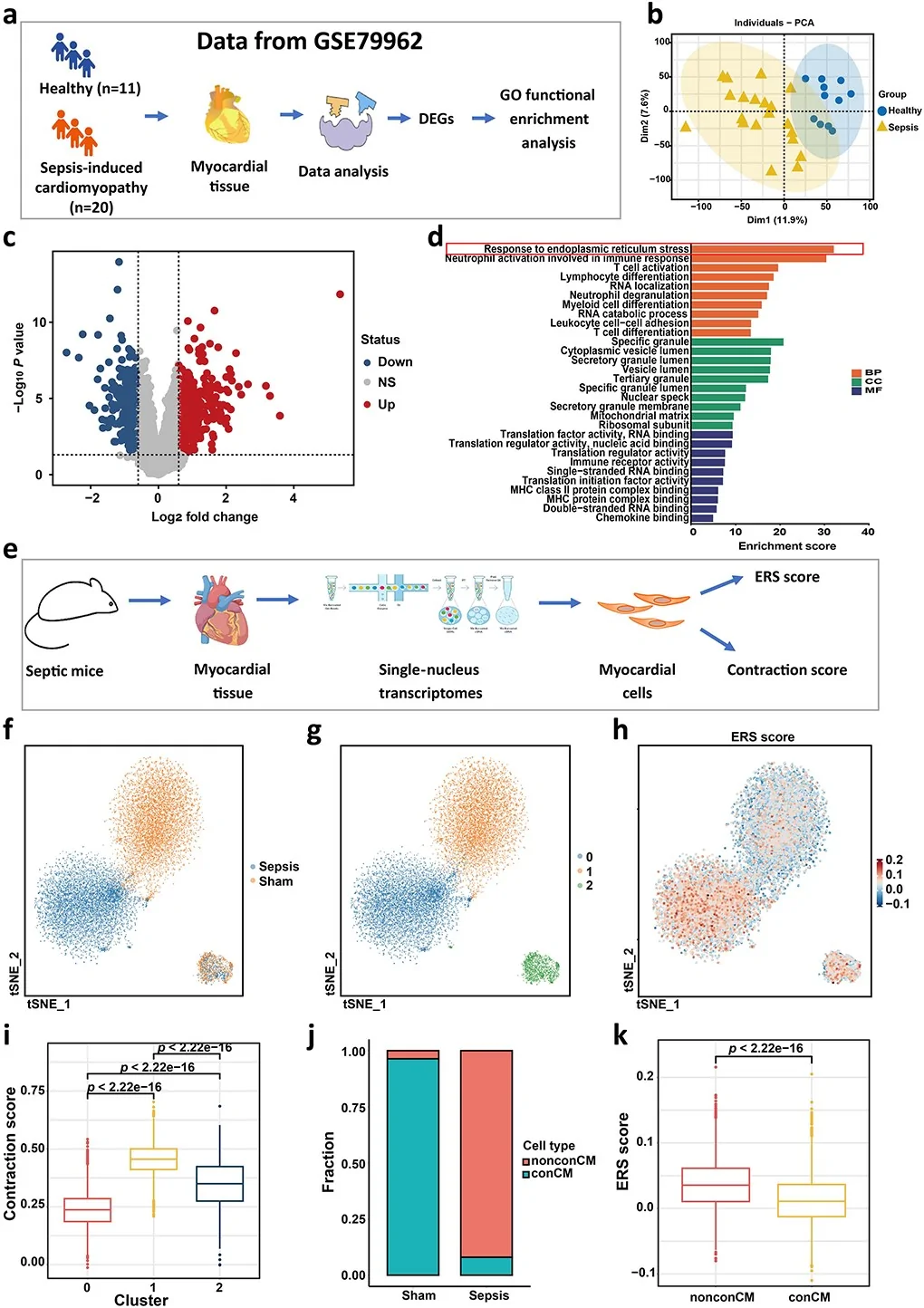
The relationship between endoplasmic reticulum stress and myocardial contraction in sepsis mice. (**a**) Bioinformatic analysis workflow of the GSE79962 dataset. The bioinformatic analysis pipeline for identifying DEGs in sepsis-induced cardiomyopathy. Gene expression data were obtained from the GSE79962 dataset, including 11 healthy control subjects and 20 patients with sepsis-induced cardiomyopathy. Myocardial tissue samples were analyzed to identify DEGs, followed by GO functional enrichment analysis to explore the BPs and molecular functions (MFs) involved in the pathogenesis of sepsis-induced cardiac dysfunction; (**b**) PCA of patients with septic cardiomyopathy and healthy individuals; (**c**) volcano plot of differential expressed genes; (**d**) GO analysis: BP, cellular component (CC) and MF; (**e**) schematic of the single-nucleus transcriptomic analysis workflow in septic mice. The experimental pipeline for snRNA-seq analysis in a murine model of sepsis-induced cardiomyopathy. Briefly, myocardial tissue was harvested from septic mice, and single-nucleus transcriptomes were profiled to identify cell subpopulations. Cardiomyocytes were then extracted for downstream analysis, where ERS scores and myocardial contraction scores were calculated to link ERS activation with cardiac contractile function. (**f**) t-SNE plot of the sepsis and the control group (*n* = 3); (**g**) t-SNE plot of different cell populations; (**h**) t-SNE plot of the ERS scores of different cell subsets; (**i**) box plot of the contraction scores of different cell populations; (**j**) proportion of contractile and non-contractile cardiomyocytes in the sham group and the sepsis group; (**k**) box plot of the ERS scores in contractile and non-contractile cells. *nonconCM* non-contractile cardiomyocytes, *conCM* contractile cardiomyocytes, *Sham* Sham operation group, *ERS score* endoplasmic reticulum stress score, *Contraction score* myocardial cell contraction score

To further analyze relationship of ERS with sepsis induced cardiac damage, the transcriptome dataset (GSE79962) in myocardial tissues from sepsis patients were analyzed, the dataset included 20 sepsis samples and 11 normal samples (Figure 1a). The 674 DEGs were identified among which 373 genes were up-regulated and 301 genes were down-regulated in sepsis cardiomyopathy (Figure 1b and c; Figure S2a). GO functional enrichment analysis showed that among the upregulated DEGs, most of genes were associated with ERS, neutrophil-related processes, and T cell-mediated immune responses (Figure 1d). Above results indicate that the ERS is the major pathway related with septic cardiac dysfunction.

To further testify the relationship of ERS with cardiac dysfunction following sepsis, single-nucleus transcriptomes of myocardium in septic mouse were detection (Figure 1e). t-SNE results of single-nucleus cell transcriptomes presented significant difference in cardiomyocytes between the sham and sepsis (Figure 1f), and three subpopulations of cardiomyocytes were identified (Figure 1g). The function analysis showed that Cluster 0 was mainly associated with inflammatory and immune pathway-related genes, while Clusters 1 and 2 were primarily involved in cardiac muscle contraction and mitochondrial metabolism (Figure S2b). The analysis of relationship of ERS and cardiac dysfunction after sepsis showed that ERS scores was higher in sepsis group than sham group, at same time the contraction-related genes score was decreased following sepsis (Figure S2c and d). Among three subpopulations, cardiomyocytes from Clusters 1 and 2 presented higher myocardial contraction scores and lower ERS scores (Figure 1h and i). To further observe the relationship of ERS and contraction, cardiomyocytes were further classified into contractile and non-contractile types via contractile gene expressions. In total, 98.9% of cardiomyocytes were contractile type in sham group, while non-contractile cardiomyocytes were major type after sepsis, contractile type accounted for 10.9% (Figure 1j; Figure S2e). Notably, non-contractile cardiomyocytes exhibited significantly higher ERS scores than contractile type (Figure 1k). The results indicate that ERS may play important role in cardiac dysfunction following sepsis.

### Beneficial effect of endoplasmic reticulum stress inhibition on myocardial dysfunction after sepsis

**Figure 2:**
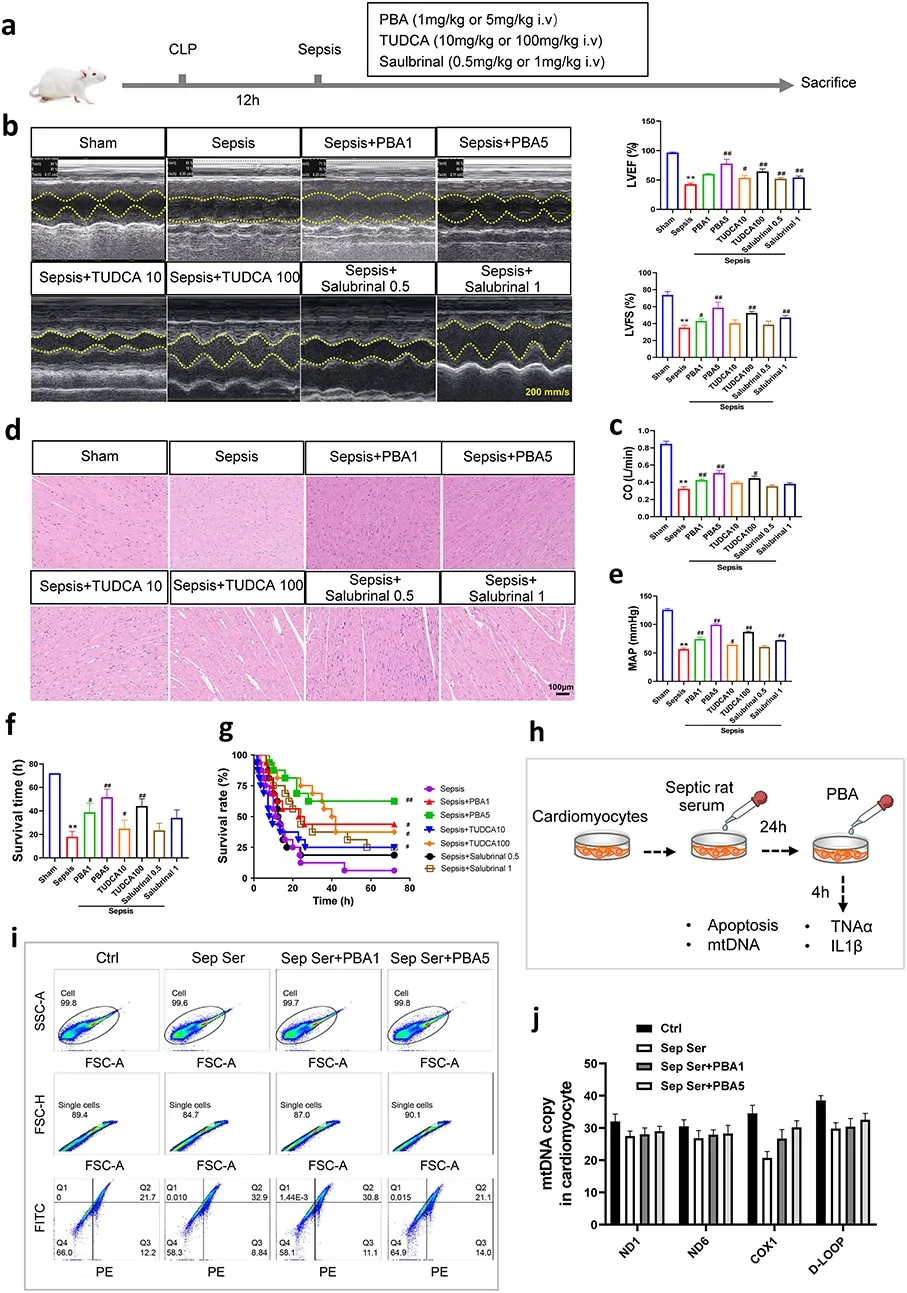
Effects of endoplasmic reticulum stress inhibitors on cardiac function of sepsis rats. (**a**) Flow chart of the intervention effects of ERS inhibitor in septic rats; (**b**) echocardiogram, LVEF of sepsis rats treated with endoplasmic reticulum stress inhibitors (*n* = 3), LVFS of sepsis rats treated with endoplasmic reticulum stress inhibitors (*n* = 3); (**c**) effects of different endoplasmic reticulum stress inhibitors on the CO (*n* = 8); (**d**) effects of different endoplasmic reticulum stress inhibitors on the myocardial pathological structure (Scale bar: 100 μm, *n* = 3); (**e**) effects of different endoplasmic reticulum stress inhibitors on the mean arterial blood pressure (MAP, *n* = 8); (**f**) effects of different endoplasmic reticulum stress inhibitors on the survival time (*n* = 16); (**g**) effects of different endoplasmic reticulum stress inhibitors on the survival rate (*n* = 16); (**h**) schematic overview of the experimental workflow. Cardiomyocytes were first treated with septic rat serum for 24 h to establish a septic injury model. Subsequently, the cells were exposed to 4-PBA for an additional 4 h. At the end of the treatment period, cells were harvested for subsequent analyses, including measurements of apoptosis, mtDNA release, and expression levels of pro-inflammatory cytokines such as tumor necrosis factor-alpha and IL-1β; (**i**) detection of apoptosis of H9C2 by flow cytometry (*n* = 3); (**j**) detection of changes in mtDNA by qPCR (*n* = 3). ^**^: *P* < 0.01, compared with the sham operation group; ^#^: *P* < 0.05, ^##^: *P* < 0.01, compared with the sepsis group. *Sepsis+PBA1* infusion treatment with 1 mg/kg of 4-PBA after sepsis, *sepsis+PBA5* infusion treatment with 5 mg/kg of 4-PBA after sepsis, *sepsis+TUDCA 10* treatment with 10 mg/kg of TUDCA after sepsis, *sepsis+TUDCA 100* treatment with 100 mg/kg of TUDCA after sepsis, *sepsis+salubrinal 0.5* treatment with 0.5 mg/kg of salubrinal after sepsis, *sepsis+salubrinal 1* treatment with 1 mg/kg of salubrinal after sepsis, *Sep ser* group stimulated by sepsis serum, *Sep ser + PBA1* 1 mmol/L of 4-PBA is added after stimulation with sepsis serum, *Sep ser + PBA5* 5 mmol/L of 4-PBA is added after stimulation with sepsis serum, *ND1* nicotinamide adenine dinucleotide dehydrogenase 1, *COX1* cytochrome C oxidase subunit 1, *D-LOOP* control region

Above results showed that ERS was closely relationship with septic cardiac dysfunction, the effects of ERS inhibition on myocardial function were investigated. The 3 types of ERS inhibitors including 4-PBA (1 and 5 mg/kg), tauroursodeoxycholic acid (TUDCA, 10 and 100 mg/kg), and salubrinal (0.5 and 1 mg/kg) were used in our study (Figure 2a). The cardiac dysfunction including LVEF, LVFS, and CO were decreased after sepsis, inhibition of ERS improved cardiac function after sepsis. (Figure 2b and c). ERS inhibition antagonized sepsis-induced myocardial fibers rupture, interstitial edema, and inflammatory cell infiltration (Figure 2d). And ERS inhibition could significantly improve the MAP and survival in septic rats (Figure 2e–g). The protective effect of 4-PBA (5 mg/kg) was best.

The pharmacokinetics effects of different 4-PBA doses including 1, 5, 10, 20, and 50 mg/kg were observed. The results showed that protective effects in 5,10, 20 mg/kg of 4-PBA were similar, while 50 mg/kg of 4-PBA administration induced damage to cardiac function (Figure S3a–i). The pharmacokinetics analysis showed that after administrations of 4-PBA (5 mg/kg), its half-life was ~5 h, with plasma concentrations of 8.20–10.30 mg/L at 4 h, which is consistent with the therapeutic effect (Figure S3j–m, Tables S1–S8). These results indicate that inhibition of ERS plays significant protective role in septic cardiac function, the effect of 4-PBA is best.

Previous studies found that ERS induced apoptosis and inflammation, the influence of 4-PBA on inflammation activation and apoptosis in cardiomyocyte were investigated. H9C2 cells were incubation with 10% septic rat serum for 24 h, 1 or 5 mmol/L of 4-PBA were followed for 4 h. The results showed that 4-PBA significantly lightened sepsis serum incubation-induced apoptosis in H9C2 (Figure 2h and i). At the same time, 4-PBA reduced sepsis serum-induced the increase of TNFα and IL-1β in H9C2 (Figure S3n and o). Furthermore, the mtDNA level was assessed via PCR by CT values of ND1, ND6, COX1, and D-LOOP regions. The result showed that sepsis serum led to mtDNA release, it was expressed with the decrease of CT values of above markers, 4-PBA administration significantly reduced extracellular mtDNA release (Figure 2j).

### The role of mitochondrial dynamic balance and mitochondria-associated endoplasmic reticulum–membrane formation in the 4-phenylbutyric acid protecting on cardiac function after sepsis

#### The role of mitochondrial dynamic in 4-phenylbutyric acid protecting sepsis-induced myocardial dysfunction

**Figure 3:**
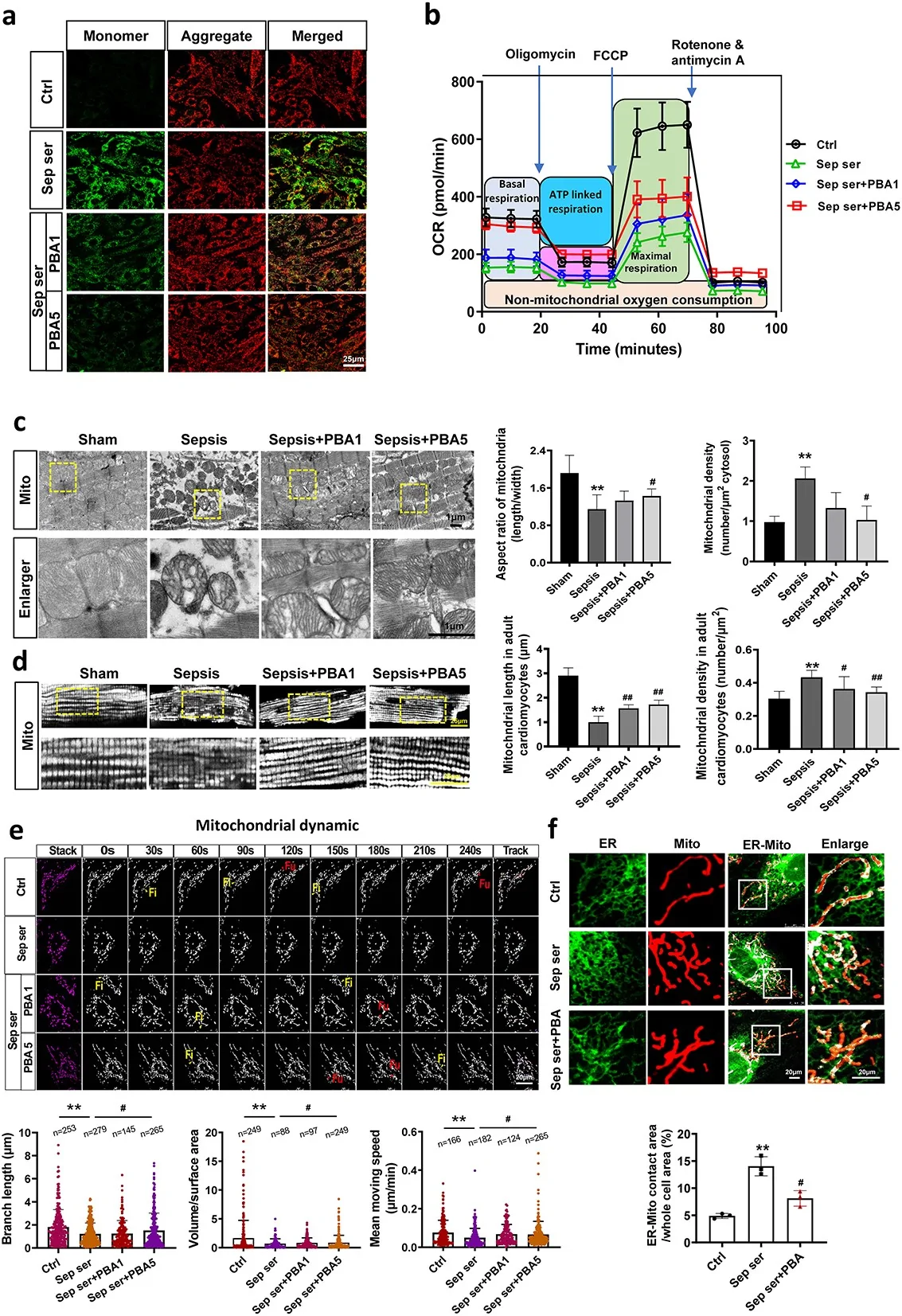
Effects of 4-PBA on the mitochondrial dynamic balance in sepsis rats and myocardial cell. (**a**) Effect of 4-PBA on mitochondrial membrane potential (∆Ψm) (Scale bar = 25 μm, *n* = 3); (**b**) effect of 4-PBA on OCR (*n* = 3); (**c**) effect of 4-PBA on myocardial mitochondria by TEM (Scale bar = 1 μm, *n* = 3); (**d**) effect of 4-PBA on mitochondrial structure from isolated cardiomyocytes of sepsis rats (Scale bar: 25 μm); (**e**) effect of 4-PBA on mitochondria by laser confocal microscopy (Scale bar: 20 μm, *n* = 3); (**f**) effect of 4-PBA on ER-Mito contact. ^**^: *P* < 0.01, compared with the sham operation group (the control group); ^#^: *P* < 0.05, ^##^: *P* <0.01, compared with the sepsis group (sepsis serum group). Monomer: when using JC-1 to detect the mitochondrial membrane potential, when the mitochondrial membrane potential is low, JC-1 exists in the form of monomers and shows green fluorescence. Aggregate: when the mitochondrial membrane potential is high, JC-1 enters the mitochondria and forms aggregates, showing red fluorescence. *Mito* mitochondria, S*ham* sham operation group, *sepsis+PBA1* infusion treatment with 1 mg/kg of 4-PBA after sepsis, *sepsis+PBA5* infusion treatment with 5 mg/kg of 4-PBA after sepsis, *Sep ser* group stimulated by sepsis serum, *Sep ser + PBA1* 1 mmol/L of 4-PBA is added after stimulation with sepsis serum, *Sep ser + PBA5* 5 mmol/L of 4-PBA is added after stimulation with sepsis serum

Our previous studies demonstrated that mitochondrial dynamics imbalance and its related mitochondrial dysfunction were critical for in the myocardial dysfunction after sepsis, effects of 4-PBA on mitochondrial dynamics and its function were investigated. The results showed that mitochondrial function were markedly disturbed in cardiomyocytes (H9C2) after sepsis, which characterized with the decreases of mitochondrial membrane potential (Figure 3a; Figure S4a), OCR (Figure 3b; Figure S4b) and ATP production (Figure S4c), and the increase of reactive oxygen species (ROS) level (Figure S4d). 4-PBA (1 mM or 5 mM) significantly improved the mitochondrial function.

In myocardial tissue, the mitochondria were orderly distributed along the myofibrils with an aspect ratio of 1.71 and an area density of 0.97μm^2^ in normal rats by transmission electron microscopy (TEM). Following sepsis, the mitochondrial arrangement was disordered and swollen, and mitochondrial cristae were disappeared, the volume of mitochondria was smaller and numbers was increased with an aspect ratio of 1.38 and the density of 1.86 μm^2^. 4-PBA administration significantly improved the mitochondrial structure with the increase of an aspect ratio and the reduce of the mitochondrial density in myocardial tissue (Figure 3c).

In acutely isolated myocardial cells from rats, mitochondria were cylindrical and orderly arranged in normal condition by confocal laser microscopy. In myocardial cells from septic rat, the arrangement of mitochondria was disordered with smaller volume. 4-PBA improved mitochondrial structure with uniform arrangement and intact morphology (Figure 3d).

In H9C2 cell, sepsis led to mitochondrial dynamics imbalance with the reduce of branch length of mitochondria and the increase of punctate mitochondria number by time-lapse imaging. 4-PBA improved mitochondrial dynamics balance with the increase of mitochondrial branch length and the alleviation of mitochondrial fragmentation. In addition to mitochondrial branch length, sepsis reduced the volume ratio of mitochondria to whole cells and mitochondrial movement speed. 4-PBA increased the volume ratio of mitochondria and the mitochondrial movement speed in myocardial cells after sepsis (Figure 3e). Above results suggest that 4-PBA improve mitochondrial function by restoring mitochondrial dynamics in myocardial cells and then improve cardiac function for sepsis.

#### 4-phenylbutyric acid recovered mitochondrial dynamic balance via decreasing the formation of mitochondria associated endoplasmic reticulum–membrane

Sepsis triggered the translocation of mitochondrial fission protein DRP1 from the cytoplasm to mitochondria, which was consistent with previous findings. Meanwhile, sepsis led to the decrease of the mitochondrial fusion proteins MFN2 and OPA1 expressions. However, 4-PBA exerted no obvious effects on sepsis-induced DRP1 translocation, as well as the expression levels of MFN2 and OPA1 (Figure S5a–e). These results indicate that 4-PBA recovering mitochondrial dynamics balance does not related with the expression and distribution of mitochondrial dynamic proteins, other mechanisms may be involved in the 4-PBA recovering mitochondrial dynamics balance.

Base on ER wrapping mitochondria inducing mitochondrial pre-constriction plays critical role in mitochondrial fission. The MAM formation in myocardial cells (H9C2 cells) was observed. These data showed that the formation of MAM was notably increased after sepsis (white), as evidenced by colocalization with ER (green) and mitochondria (red). The overlap coefficient, the area ratio between MAM (white signal) to the total area of the ER and mitochondria (red and green signal), was increased by 3.1-fold after sepsis serum stimulation as compared with control group. 4-PBA (5 mM) treatment significantly reduced the overlap coefficient, decreasing by 36.1% compared to sepsis serum group (Figure 3f). These results suggest that 4-PBA recover mitochondrial dynamics balance via reducing the MAM formation.

### Lactate participated in the 4-phenylbutyric acid reducing mitochondria associated endoplasmic reticulum–membrane formation and recovering the mitochondrial dynamic balance in cardiomyocytes

#### 4-PBA led to metabolic reprogramming and reduce the lactate level in cardiomyocytes

**Figure 4:**
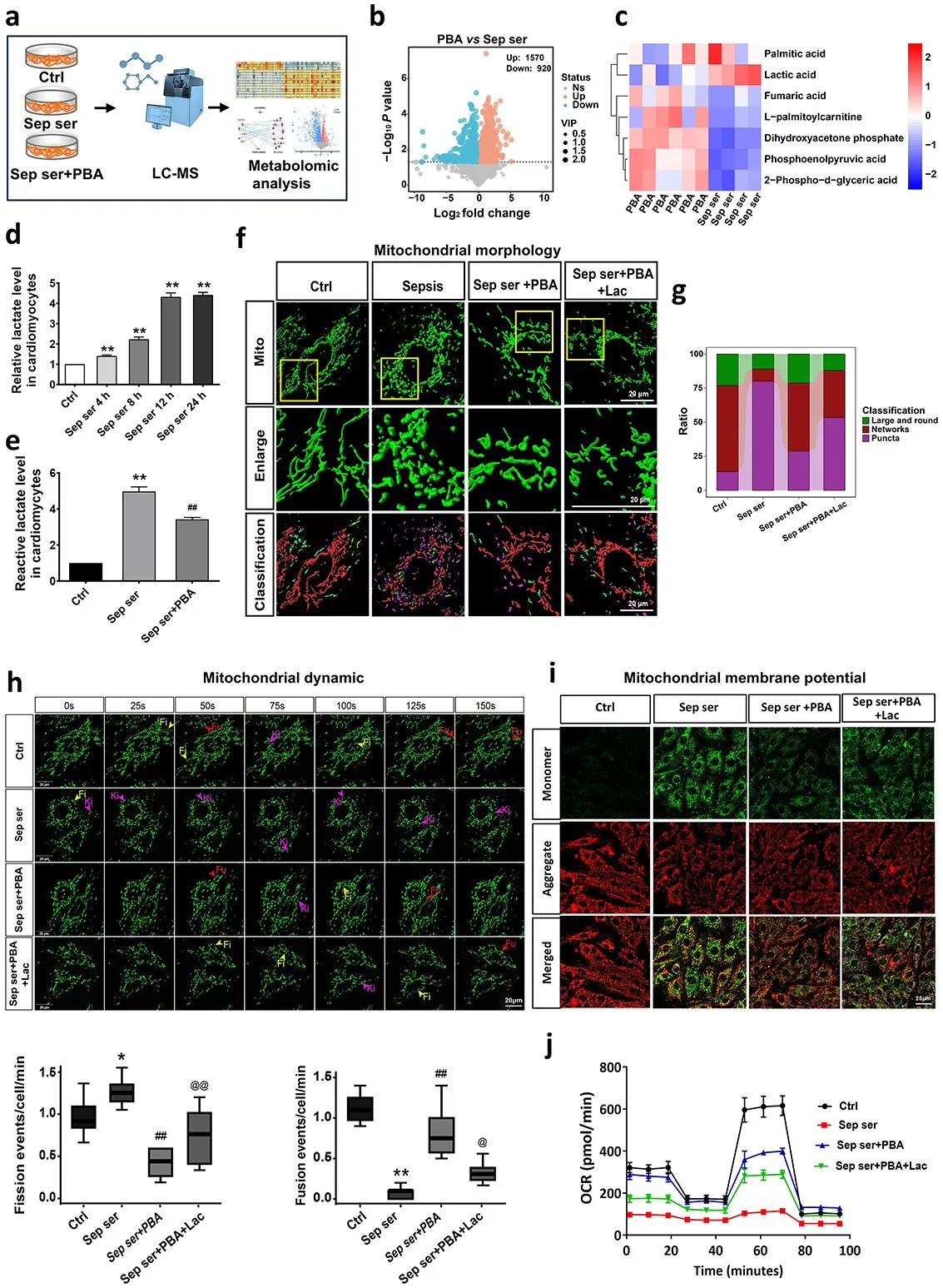
Role of lactic acid in the regulation of mitochondrial dynamic and mitochondrial function in cardiomyocytes (H9C2) treated with sepsis serum and 4-PBA. (**a**) Schematic diagram of the flow-chart for metabolomics analysis of H9C2 cells. (**b**) Volcano plot of differential metabolites between the 4-PBA group and the sepsis serum group. The scatter points represent metabolites. The horizontal axis is the logarithmic value of the FC, and the vertical axis is the negative logarithmic value of the *P*-value with base 10. Red, blue, and gray represent significantly up-regulated, down-regulated, and non-statistically-significant metabolites, respectively. (**c**) Cluster heatmap of differential metabolites between the 4-PBA group and the sepsis serum stimulation group. Red and blue represent high and low expression, respectively. (**d**) Lactate levels at different time points of sepsis serum stimulation (*n* = 8). (**e**) The effect of 4-PBA on lactate levels (*n* = 8). (**f**) The morphology of mitochondria after lactate treatment (bar = 20 μm, *n* = 3); (**g**) statistics of mitochondrial morphology after lactate treatment. (**h**) Time-lapse results of mitochondrial movement after lactate treatment; (**i**) effect of lactate on mitochondrial membrane potential (∆Ψm) (bar = 25 μm, *n* = 3); (**j**) effect of lactate on OCR (*n* = 3). ^**^: *P* < 0.01, compared with ctrl group; ^#^: *P* < 0.05, ^##^: *P* < 0.01, compared with the sepsis serum stimulation group. @: *P* < 0.05, @@: *P* < 0.01, compared with 4-PBA. *Sep ser* group stimulated by sepsis serum, *sep ser + PBA* 5 mmol/L of 4-PBA is added after stimulation with sepsis serum, *Sep ser + PBA + lac* 5 mmol/L of 4-PBA and 10 mm lactate are added after stimulation with sepsis serum, *lac* lactate

Several mechanisms took part in the sepsis-related cardiac dysfunction, metabolic disorder was a key contributor [11]. As one of the most metabolically active organs, the heart was highly sensitive to septic metabolic stress. To evaluate mechanisms of 4-PBA protecting cardiac function, the untargeted metabolomic profiling were performed in H9C2 cells (Figure 4a). The results showed that sepsis serum induced the metabolic profile disturbance, while 4-PBA (5 mM) antagonized the sepsis-induced metabolic status in myocardial cells (Figure S6a and b). As compared with sepsis serum group, a total of 1520 metabolites were elevated and 920 were suppressed after 4-PBA administration in myocardial cells (with a 1.5-FC set as the threshold for significant upregulation and a value below 1/1.5 as the threshold for significant downregulation). Sepsis-induced glycolysis was inhibited significantly by 4-PBA in myocardial cells, and lactate levels was reversed by 4-PBA (Figure 4b and c). The changes of lactate levels following sepsis and 4-PBA treatment were testified by experimental detection (Figure 4d and e).

#### Lactate participated in the 4-phenylbutyric acid alleviating mitochondria associated endoplasmic reticulum–membrane formation in cardiomyocytes

Above results showed that 4-PBA recovered mitochondrial dynamic balance via reducing MAM formation, moreover 4-PBA inhibited sepsis-induced lactate accumulation. The relationship of lactate with the MAM formation in H9C2 cells was investigated. The results showed that lactate (10 mmol/L) incubation abolished the effect of 4-PBA on the formation of MAM (Figure S7a). Mitochondrial dynamics measurements showed that mitochondria structure and network were disrupted after sepsis with 80% of punctate mitochondria. 4-PBA (5 mM) treatment recovered the mitochondrial structure and network and reduced punctate mitochondria ratio. Lactate incubation canceled effects of 4-PBA on mitochondria morphology (Figure 4f and g). Live-cell Time-Lapse imaging (yellow arrows indicate fission, red arrows indicate fusion) showed that the frequencies of fission and fusion kept balance in myocardial cells in normal group, while frequency mitochondrial fission was increased by ~44.8% after sepsis and frequency of mitochondrial fusion was decreased. 4-PBA recovered mitochondrial dynamic balance significantly, lessening fission frequency and enhancing fusion. Lactate incubation antagonized 4-PBA on mitochondrial dynamics (Figure 4h). Consistently with mitochondrial dynamic balance, lactate abolished 4-PBA’s improvement on mitochondrial function in H9C2 cells (Figure 4i and j; Figure S7b–e). These results indicate that lactate take part in the 4-PBA decreasing MAM formation and improving mitochondrial dynamic imbalance in sepsis.

#### The role of knockout of MCT1 in 4-phenylbutyric acid recovering mitochondrial dynamics and mitochondrial function in septic myocardium

**Figure 5:**
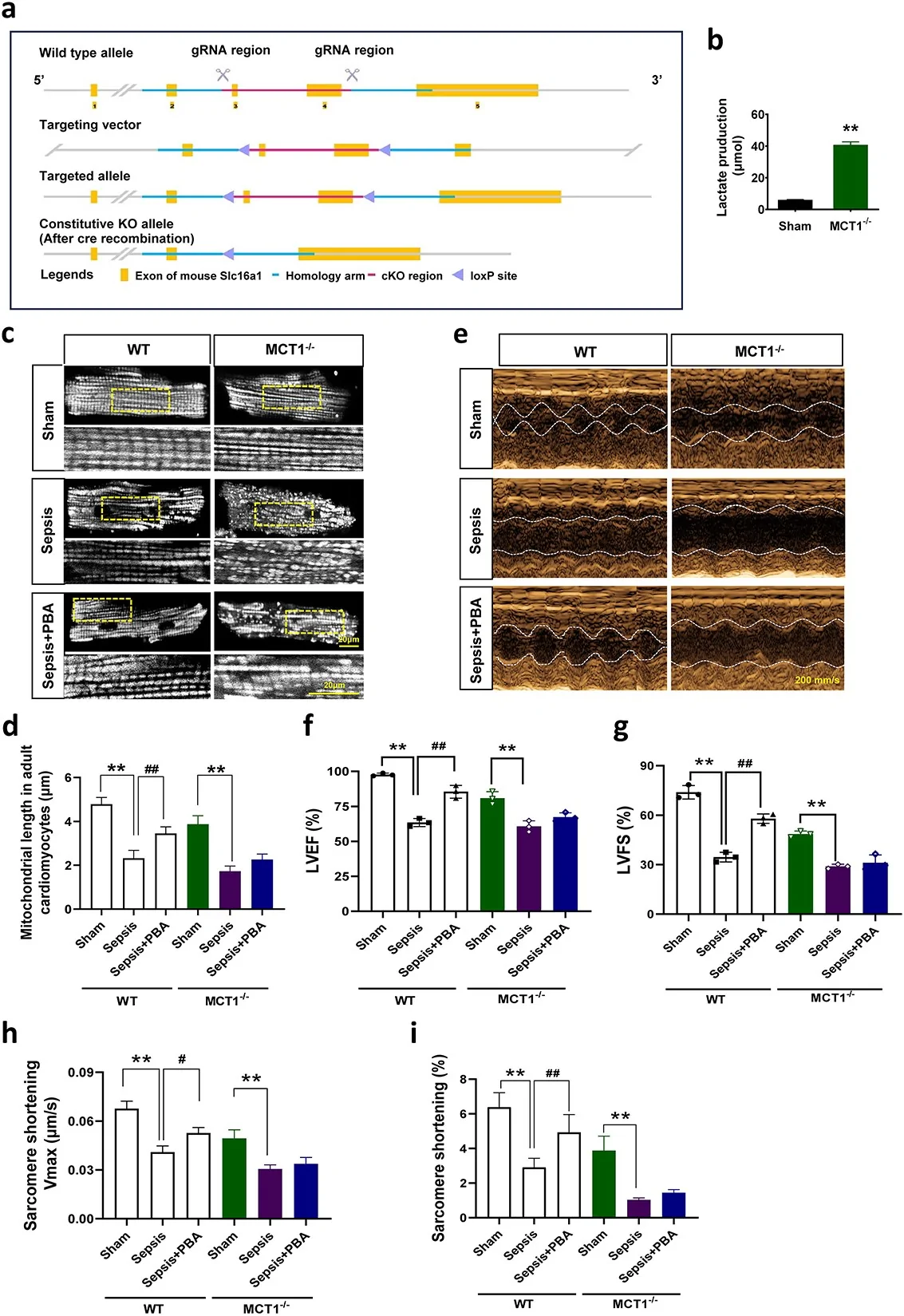
Effects of knockout MCT1 on mitochondrial dynamics, mitochondrial function and heart function in septic mice. (**a**) Strategy for knocking out the gene slc16a1 (MCT1); (**b**) effects of MCT1 knockout on the lactate level (*n* = 8). (**c** and **d**) Effects of MCT1 knockout on the mitochondrial morphology of acutely isolated cardiomyocytes (*n* = 8). (**e**) Representative echocardiography images in septic wild-type and MCT1-knockout rats with or without PBA intervention. (**f**) Quantitative analysis of LVEF in septic wild-type and MCT1-knockout rats with or without PBA intervention (*n* = 3). (**g**) Quantitative analysis of LVFS in septic wild-type and MCT1-knockout rats with or without PBA intervention (*n* = 3). (**h**) Effects of MCT1 knockout on sarcomere shortening amplitude (*n* = 8). (**i**) Effects of MCT1 knockout on maximum sarcomere shortening amplitude (*n* = 8). ^**^: *P* < 0.01, compared with the sham group; ^##^: *P* < 0.01, compared with the sepsis group. *MCT1* monocarboxylate transporter 1

To further verify the impact of lactate on 4-PBA on mitochondrial dynamics balance, the cardiomyocyte specific MCT1-KO mice to increase lactate level was construct. The efficiency of MCT1-KO and the changes of lactate levels were validated (Figure 5a and b; Figure S8a). Mitochondrial dynamics were imbalanced in cardiomyocytes of MCT1-KO mice, characterized by smaller and shorter mitochondria. Sepsis further exacerbated mitochondrial dynamic imbalance, with significantly shortened mitochondria. 4-PBA (5 mg/kg) failed to restore mitochondrial dynamic balance in cardiomyocytes in MCT1-KO mice (Figure 5c and d). Mitochondrial function assays showed that MCT1-KO induced mitochondrial dysfunction, with the reduce of ATP content and the increase of ROS levels in cardiac tissues. 4-PBA could not improve MCT1-KO-induced mitochondrial dysfunction (Figure S8b and c). 4-PBA improved sepsis-induced cardiac dysfunction including the decrease of LVEF, LVFS and sarcomere contraction responses to electrical stimulation, while 4-PBA failed to 4-PBA failed to restore LVEF, LVFS and sarcomere contraction responses in septic MCT1-KO mice (Figure 5e–i). The results indicate that lactate participate in the protective roles of 4-PBA on mitochondrial dynamics and cardiac function in sepsis.

### Lactate related Arpc1b lactylation played important role in the mitochondria associated endoplasmic reticulum–membrane formation and mitochondrial dynamic balance in cardiomyocytes after sepsis

#### 4-phenylbutyric acid lessened protein lactylation level via reducing lactate level in cardiomyocytes

**Figure 6:**
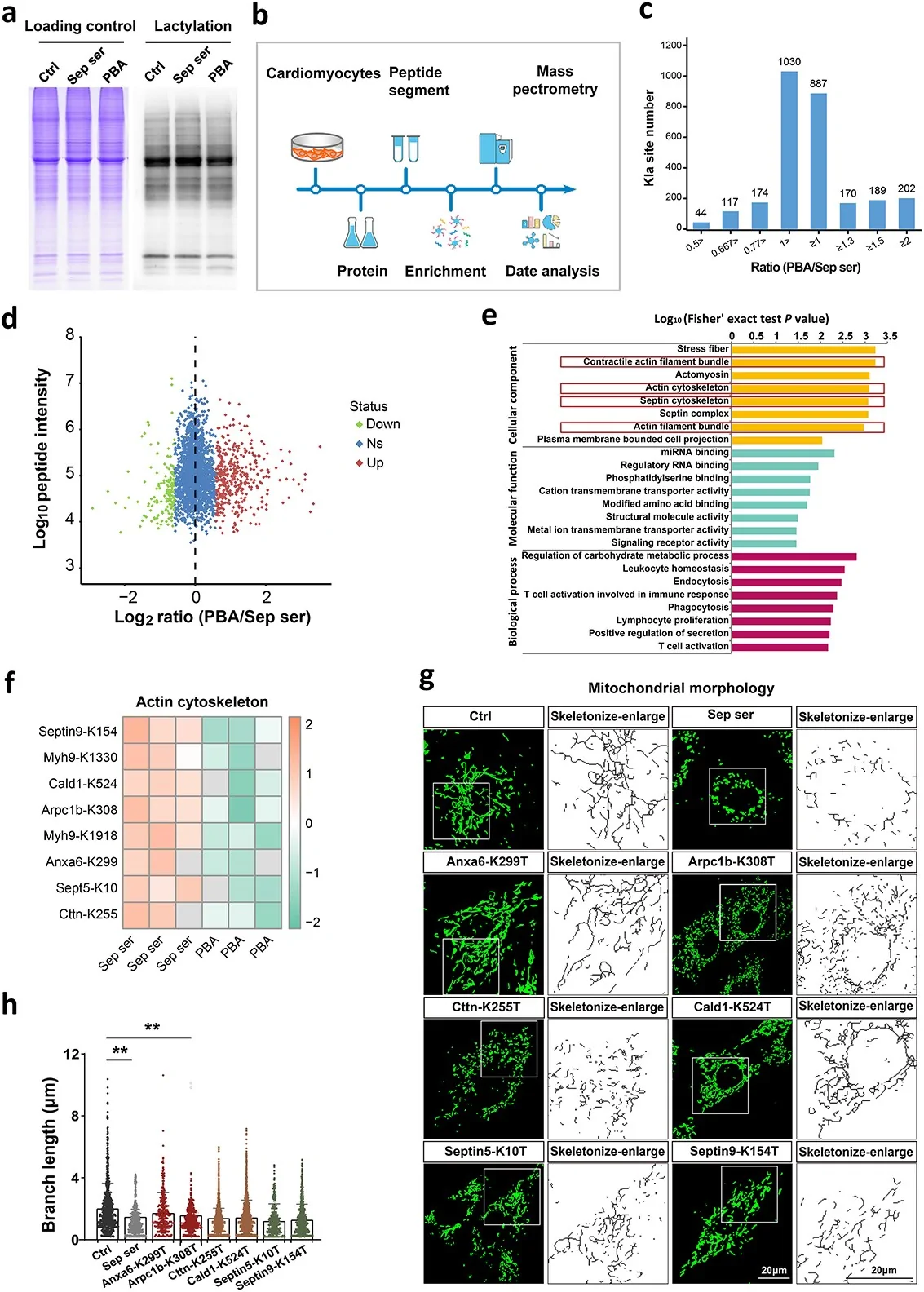
Effects of skeletal proteins lactylation on mitochondrial morphology in cardiomyocytes (H9C2). (**a**) Lactylation of total proteins in myocardial cells (*n* = 3). (**b**) Flow chart of lactylation modification omics (*n* = 3). (**c**) The differential distribution of lactylation sites between the 4-PBA group and the sepsis group. (**d**) Scatter plot of the ion intensity of lactylated peptides. (**e**) GO enrichment analysis of proteins with differentially modified sites; (**f**) DEPs and their modified sites in the cytoskeleton enriched by GO analysis. (**g** and **h**) The effects of mutant plasmids of Arpc1b-K308, Anxa6-K299, Cald1-K524, Cttn-K255, Septin5-K10, and Septin9-K154 on mitochondrial morphology (Scale bar: 20 μm, *n* = 3). *Sep ser* stimulated by sepsis serum, *Sep ser + PBA* 5 mmol/L of 4-PBA is added after stimulation with sepsis serum

Lactate could serve as a substrate for protein post-translational modification, bridging metabolism and protein function [12]. The protein lactylation level was measured following 4-PBA treatment, detection with pan-antibody revealed that the total protein lactylation level was increased in cardiomyocytes (H9C2 cells) after sepsis serum stimulation, and 4-PBA (5 mM) decreased the total lactylation level of proteins (Figure 6a). The acetylation proteomics showed that 1649 proteins were lactylated after sepsis, among which 46% (758) of the proteins had only one modification site, and 20% (324) had two modification sites (Figure 6b and c; Figure S9a and b). 4-PBA treatment resulted in 117 sites with down-regulated lactylation in cardiomyocyte proteins as compared with sepsis group. A 1.5-FC was set as the cutoff: values over 1.5 indicated significant upregulation, and values below 1/1.5 indicated significant downregulation (Figure 6d). GO functional enrichment analysis showed the proteins differentially modified sites were mainly involving in cytoskeletal proteins, such as actin cytoskeleton, septin cytoskeleton, and other pathways (Figure 6e).

Based on the effects of actin on the formation of MAM, the lactylation levels of cytoskeletal proteins were analyzed. The results showed that several cytoskeletons proteins were lactylated including Arpc1b-K308, Anxa6-K299, Cald1-K524, Cttn-K255, Septin5-K10, and Septin9-K154 after sepsis, 4-PBA treatment significantly reduced their lactylation (Figure 6f). To screen which cytoskeletal protein played the critical role in regulating MAM formation, the lactylation sites of the above proteins were mimicked by site-directed mutagenesis. The results showed that lactylation-mimetic mutation of Arpc1b-K308 and Cttn-K255 induced the increase of MAM formation, and the effects of lactylation- mimetic mutation of Arpc1b-K308 were better (Figure S10a and b). As Arp2/3 modulating subunit, Arpc1b controlled actin bundling and meshwork formation. The following studies showed that lactylation-mimetic mutation of Arpc1b-K308 led to mitochondrial fragmentation and mitochondria presented as puncta with shortened branch lengths similar to sepsis (Figure 6g and h).

#### Arpc1b lactylation plays important role in mitochondria associated endoplasmic reticulum–membrane formation and mitochondrial dynamics in myocardial cells

**Figure 7:**
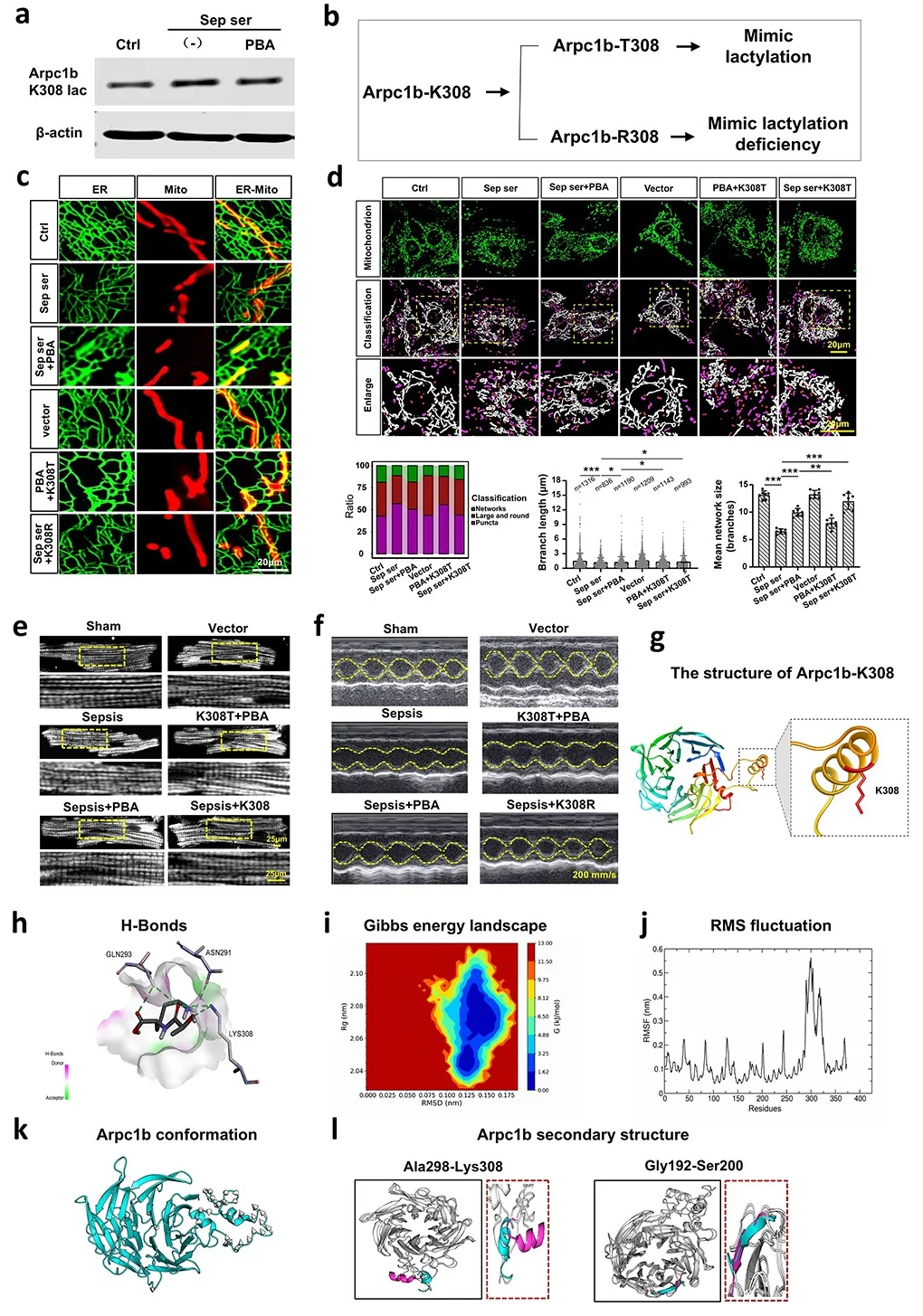
Role of lactylation modification of Arpc1b-K308 in H9C2 cells and heart function. (**a**) Western blot analysis of the changes in the lactylation of Arpc1b-K308 in H9C2 cells after the treatment with 4-PBA (*n* = 3); (**b**) schematic of Arpc1b site-specific lactylation mimicry constructs. The generation of site-specific Arpc1b mutants to mimic or abolish lactylation at residue 308. The wild-type lysine (K308) was substituted to threonine (T308) to mimic lactylation, or to arginine (R308) to mimic lactylation deficiency, enabling functional analysis of Arpc1b lactylation in the context of sepsis-induced cardiac dysfunction. (**c**) Effect of lactylation modification at lysine 308 site of Arpc1b on the formation of MAM (*n* = 3); (**d**) effect of lactylation modification at lysine 308 site of Arpc1b on the morphology of mitochondria in cardiomyocytes (H9C2 cells) (*n* = 3); (**e**) effect of Arpc1b-K308 lactylation on mitochondrial structure in cardiomyocytes isolated from mouse myocardial tissue; (**f**) effect of Arpc1b-K308 lactylation on mouse cardiac contractile function; (**g**) structure of Arpc1b-K308; (**h**) hydrogen bonds formed between the ligand and the Lys308 site; (**i**) Gibbs free energy landscape during the binding process of small molecules to the protein; (**j**) RMSF of Arpc1b; (**k**) conformational changes of Arpc1b during simulation; (**l**) dynamic changes in the secondary structure of Arpc1b protein. Blue represents the structural state at the start of simulation (0 ns), and red corresponds to the structure at the end of simulation (100 ns). ^*^: *P* < 0.05, ^**^: *P* <0.01, ^**^: *P* < 0.001. *Sep ser* stimulated by sepsis serum

To further explore the role of Arpc1b lactylation on MAM formation, the specific lactylation antibody against Arpc1b-K308 was synthesized. Results showed that lactylation modification levels of Arpc1b-K308 was increased significantly in cardiomyocytes following sepsis serum incubation, 4-PBA reduced the level of Arpc1b-K308 lactylation (Figure 7a; Figure S11a). Furthermore, K308T of Arpc1b was constructed which lysine was mutated to threonine to mimic lactylation modification, and K308R of Arpc1b was constructed which lysine mutated to arginine to mimic de-lactylation modification (Figure 7b). At same time, site-mutant cell lines (Figure S11b and c) and Arpc1b gene K308T or K308R mutants were constructed. The results showed that Arpc1b K308T site mutation (mimicking lactylation modification) significantly increased the formation of MAM, while K308R (mimicking non-lactylation modification) reduced sepsis-induced MAM increased in cardiomyocytes (Figure 7c; Figure S12a).

Mitochondrial structure observation showed that mitochondrial branch length with Arpc1b K308T site mutation in H9C2 cells exhibited a similar profile to that in the sepsis group with mitochondria fragments; K308R site mutation antagonized sepsis-induced mitochondrial fragments. The trend of mitochondrial network size was consistent with mitochondrial branch length (Figure 7d). Mitochondrial function showed similar trend of mitochondrial dynamics (Figure S12b and c).

To further testify the role of Arpc1b-K308 lactylation *in vivo*, mice were administrated with AAV9-cTNT-mArpc1b(K308R)-3Flag-P2A-EGFP (mimicking de-lactylation modification), AAV9-cTNT-mArpc1b (K308T-3Flag-P2A-EGFP (mimicking de-lactylation modification), or AAV9-cTNT-mArpc1b-3Flag-P2A-EGFP and AAV9-cTNT-mArpc1b-WT as control, specific expression of Arpc1b in heart tissue was confirmed by immunofluorescence staining (Figure S13a). The results showed that the K308R mutant improved mitochondrial morphology (increased aspect ratio, decreased density) (Figure 7e; Figure S13b and c) by mimicking de-lactylation modification, and enhanced mitochondrial function (increased ATP production, decreased ROS levels, Figure S13d and e). And the K308R mutant significantly improved cardiac systolic function and cardiac structure (Figure 7f; Figure S13f–h). K308T mutant mimicking delactylation modification play opposite roles. Above results indicated that lactate induced Arpc1b lactylation played important role in the 4-PBA restored mitochondrial dynamic balance and mitochondrial function.

#### Docking analysis of lactate induced Arpc1b K308 lactylation

Molecular docking of Arpc1b-K308 with lactyl group was performed with AutoDock, followed by binding simulation analysis with PyMOL and MOE. Results showed that Arpc1b-K308 specifically binds to the lactyl group via stable hydrogen bonds (Figure 7g and h). Potential map analysis of the binding site identified the structure with the highest binding stability between Arpc1b-K308 and lactyl group (Figure 7i). MD simulations lasting 100 ns were run in GROMACS for analyzing RMSD, RMSF and binding free energy of lactylated Arpc1b-K308. The complex showed moderate stability, but modified amino acids exhibited drastic conformational changes (Figure 7j). Structural variations were detected from 0 ns to 100 ns (Figure 7k), particularly in the Ala298-Lys308 and Gly192-Ser200 segments. Structural analysis revealed that lactylation transformed the α-helix and β-sheet structures of Arpc1b into random coils (Figure 7l).

### Hexokinase 2 played key role in the 4-phenylbutyric acid reducing lactate levels in cardiomyocytes following sepsis

**Figure 8:**
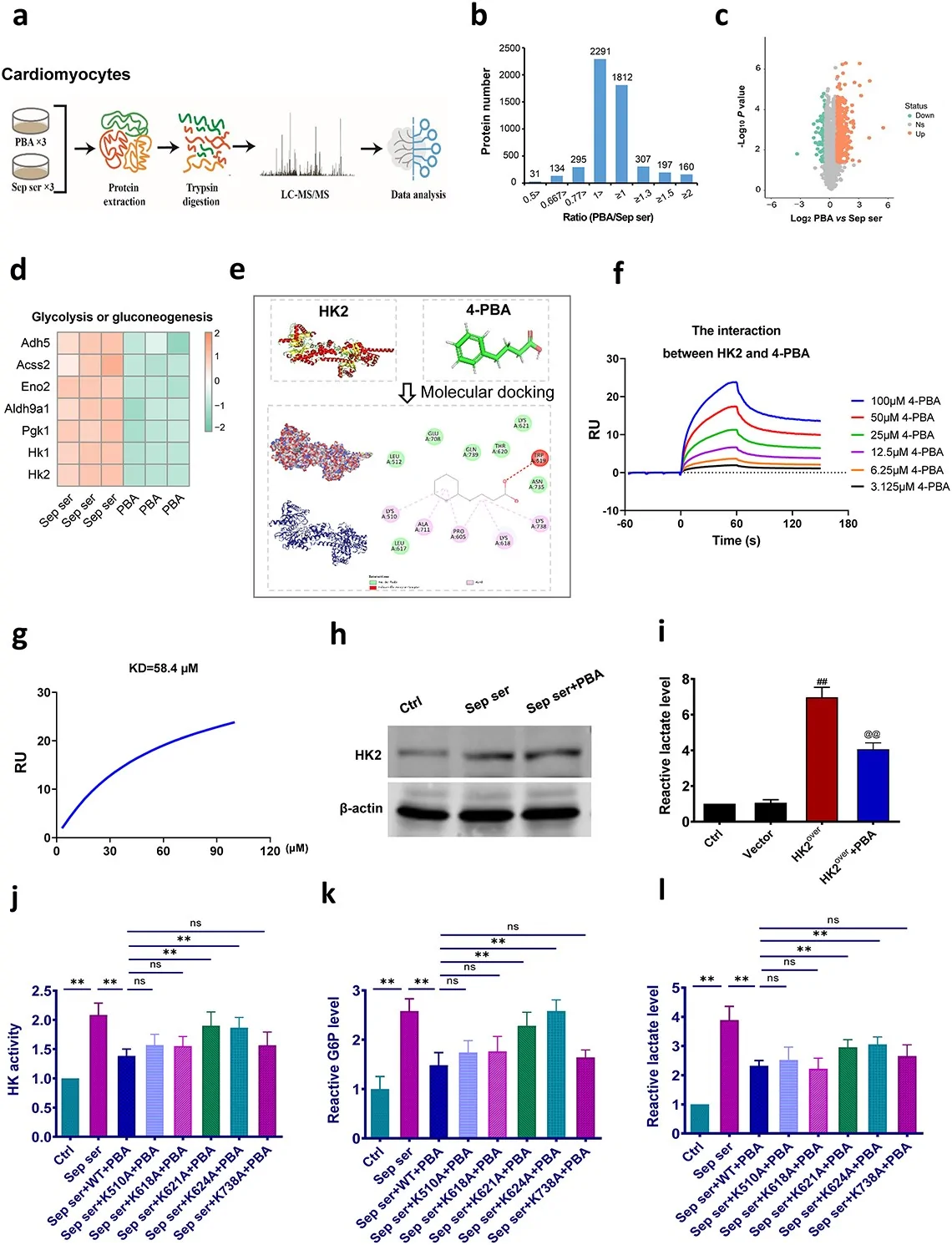
The role and mechanism of HK2 in 4-PBA-mediated regulation of lactate metabolism. (**a**) Experimental workflow of proteomics. Protein extraction, trypsin digestion, LC–MS/MS analysis, and data analysis were carried out on samples treated with 4-PBA and sepsis serum samples (*n* = 3); (**b**) bar chart for statistical analysis of DEPs; (**c**) volcano plots. These plots display the significance of differences and FCs in protein expression between the 4-PBA group and the sepsis group; (**d**) the heatmap shows the expression levels of relevant genes in the glycolysis/gluconeogenesis pathway. The intensity of the color represents the level of expression; (**e**) the three-dimensional structure of HK2 protein is shown, along with the possible binding mode between 4-PBA and HK2; (**f**-**g**) *in vitro* binding affinity between 4-PBA and HK2 protein was measured by surface plasmon resonance (SPR); (**h**) the expression of HK2 protein was analyzed by Western blotting (*n* = 3); (**i**) the changes in lactic acid levels (*n* = 8); (**j**) effect of lysine mutations at the key catalytic residues of HK2 on HK activity; (**k**) effect of lysine mutations at the key catalytic residues of HK2 on G6P levels/activity; (**l**) effect of lysine mutations at the key catalytic residues of HK2 on cellular lactate accumulation. ^##^: *P* < 0.01, compared with the sepsis serum group; @@: *P* < 0.01, compared with the 4-PBA treatment group; ^**^: *P* < 0.01; ns: no significant difference

To explore how 4-PBA reduced lactate levels, proteomic profiling of H9C2 cells was carried out to clarify the mechanism underlying lactate reduction by 4-PBA. In total, 413 proteins showed significant differential expression (*P* < .05, FC >1.5 or ≤ 0.67). Compared with the sepsis group, 4-PBA administration up-regulated 292 proteins and down-regulated 121 proteins (Figure 8a–c, Figure S14a and b). Gene set enrichment analysis (GSEA) showed notable enrichment of glycolysis/gluconeogenesis, pentose phosphate, and pyruvate pathways (Figure S14c and d). Clustering heatmap results showed that glycolytic rate-limiting enzyme HK2 was markedly downregulated after 4-PBA treatment (Figure 8d). Molecular docking showed that 4-PBA interacted with HK2 via hydrogen bonding and van der Waals forces interactions (Figure 8e). The binding affinity between 4-PBA and HK2 protein was assessed via surface plasmon resonance (SPR) *in vitro*. The results showed that the dissociation constant (KD) between 4-PBA and HK2 was 58.4 μmol/L (Figure 8f and g), which indicate a direct physical interaction between the two molecules. Western blot confirmed sepsis induced the enforcement of HK2 expression and antagonization of 4-PBA in H9C2 cells (Figure 8h; Figure S14e). The HK2 overexpression plasmids were transfected in cardiomyocyte. Results showed that HK2 overexpression increased lactate level, while 4-PBA treatment significantly reduced lactate levels compared with the HK2 overexpression group (Figure 8i). These findings demonstrate that 4-PBA reduces lactate levels by downregulating HK2 expression.

Enzyme activity was major mechanism of HK, to further explore mechanisms of 4-PBA on HK2, K510A, K618A, K621A, K624A, and K738A were key catalytic sites which are critical residues within the ATP-binding/catalytic core domain. Mutation of key catalytic sites of HK2 were constructed. The results showed HK activity, G6P levels, and lactate concentrations were markedly decreased in the wild-type HK2 group. In the K510, K618A, and K738A mutant groups, 4-PBA still exerted a suppressive effect on HK activity, G6P levels, and lactate production. In contrast, the inhibitory effects of 4-PBA on these parameters were completely abolished in the K621A and K624A mutant groups, with no reduction in HK activity. Taken together, 4-PBA must occupy the K621/K624 active sites of HK2 to block its catalytic function (Figure 8j–l).

## Discussion

Present study revealed that ERS participate importantly in sepsis induced cardiac dysfunction, and 4-PBA protected cardiac function by ERS inhibition. Beside for the apoptosis and inflammation, the mechanisms of inhibition of MAM formation and improvement of mitochondrial dynamic balance of 4-PBA played critical role in the cardiac function protection. The lactate-related lactylation of Arpc1b-K308 was key pathway participating in the 4-PBA inhibiting MAM formation via glycolysis. And 4-PBA interacted with HK2 to inhibit the lactate level. The study expanded our understanding of the pathological mechanisms for sepsis-induced cardiac dysfunction and provided a novel therapeutic measure for sepsis induced cardiomyopathy.

ERS was response to stimuli. Mild and moderate ERS enhanced cellular functions, such as increasing protein synthesis and metabolism. However, excessive ERS could trigger inflammation, oxidative stress damage even cell death, leading to cellular dysfunction. Studies found that the ER interacted with multiple organelles, such as mitochondria, Golgi apparatus, lysosome, and so on. The MAM formation was involved in the modulations of Ca^2+^ and lipid exchange [13], ERS, autophagy [14], and inflammation [15], and played a key part in the regulation of mitochondrial dynamics. Numerous studies revealed that ERS led to MAM formation disorder. For instance, the formation of MAM was increased following ERS inducing by thapsigargin [16]. Previous mechanistic studies found that ERS-related molecules PERK and IRE1α were distributed at the MAM sites. PERK modulated the formation of MAM by eIF2α to regulate the Sigma1 receptor [17]. The present study found ERS was accompanied by an increase of MAM formation following sepsis. Inhibition of ERS reduced the formation of the MAM significantly. The further studies found that inhibition of ERS reduced the level of lactate, decreased the lactylation of the cytoskeletal protein Arpc1b, and then inhibited the formation of MAM.

Previous researches showed that actin exerted key effects on the formation of the MAM which actin interacted with INF2 (a formin family protein) on the ER [18]. INF2, located on the ER, recruited actin to the ER, pulling the ER around the mitochondria to facilitate the MAM formation. Our study found that Arpc1b lactylation led to the increase of actin bundling and MAM formation. The Arp2/3 complex consists of Arp2, Arp3 and five distinct subunits (Arpc1–Arpc5). Morphologically, the complex was a flat ellipsoid ~15 nm in length, 14 nm in width, and 7–10 nm in thickness [19]. Arpc1 played a crucial role in regulating the activity of the complex, and the activity of the Arp2/3 complex was completely lost in the absence of Arpc1, which highlighted the indispensable role of Arpc1 in maintaining the activity of the complex. Arpc1 regulated the activity of the Arp2/3 complex through two main mechanisms: the Arp2/3 complex connected with multiple actins and promoted the growth of side-chain actin filaments at an angle of 70°C, and as the filaments continued to lengthen, they formed a complex network-like structure, which was important for the maintenance of intracellular substance transport [20]. Arpc1, as an essential subunit of the Arp2/3 complex, interacted with Arp2 and Arp3 to inhibit the spontaneous activation of the complex, and when activating factors (e.g. WASP, etc.) bonded to Arp2/3, the conformation of Arpc1 changes, dissociated from Arp2 and Arp3, and initiated the formation of a branching actin network [21]. The unique helical structure of Arpc1 was interconnected with the β-folding structure through an irregular loop, which could directly interact with Arp3, and then inhibited the interaction between Arpc2–4 and F-actin, and ultimately realized the fine regulation of the activity of the whole Arp2/3 complex [22]. In present study, we found that the Arpc1b lysine 308 site could undergo lactylation modification, which changed the conformation of Arpc1b and caused an increase in the formation of MAM, which in turn leads to mitochondrial dynamics imbalance and mitochondrial dysfunction. Studies have shown that conserved amino acids in Arpc1 were inserted into actin mother-filament to enhance the stability of binding to actin and promoted actin filament polymerization [19]. In this study, we used MD simulations to analyze the conformation of Arpc1b before and after lactylation. The results showed that upon binding to the lactyl group, the secondary structures of the Ala298-Lys308 and Gly192-Ser200 sequences in Arpc1b were significantly altered, transitioning from α-helices and β-sheets to random coils, which enhanced actin bundling. The findings indicate that lactylation of Arpc1b might promote actin bundling by altering the structure of Arpc1b, thereby increasing the formation of MAM and influencing mitochondrial dynamics and function. However, how lactylation modification of Arpc1b regulates actin filament bundling warrants further investigation.

In the study, the effects of various ERS inhibitors were observed, and the results found that 4-PBA exhibited the most potent effect. As an aromatic carboxylic acid, 4-PBA acted as a histone deacetylase (HDAC) inhibitor to regulate gene expression [23], influencing the expression of genes related to cellular metabolism and stress responses. Previous studies have shown that in ERS-induced mitochondrial dysfunction, 4-PBA could repair mitochondrial function, enhance mitochondrial membrane potential, and alleviate hepatotoxicity [24]. Our study further showed that 4-PBA not only inhibited ERS but also reduced the MAM formation via metabolic reprogramming, maintaining mitochondrial dynamics balance and protecting against septic cardiac dysfunction. Mechanistic studies showed that 4-PBA regulated the MAM formation by inhibiting HK2 expression, thereby reducing lactate production. Mechanistic investigations revealed that 4-PBA inhibited HK2 activity, ultimately reducing lactate production. Notably, molecular docking and site-directed mutagenesis analyses further clarified the direct interaction between 4-PBA and HK2: 4-PBA binds specifically to the K621 and K624 residues within the ATP-binding/catalytic core domain of HK2, mediated by hydroxyl groups and van der Waals forces. This binding event disrupts the catalytic function of HK2, as mutation of these residues (K621A and K624A) abolished both the enhancement of glycolysis induced by HK2 overexpression and the inhibitory effects of 4-PBA on lactate production. HK2, a rate-limiting enzyme of glycolysis, catalyzes the conversion of glucose to glucose-6-phosphate (G6P), driving lactate accumulation. Beyond its metabolic role, HK2 localizes to the outer mitochondrial membrane to inhibit mitochondrial permeability transition pore opening, thereby sustaining mitochondrial function. However, aberrant HK2 activation triggers hyper-glycolysis, leading to lactate overload and subsequent mitochondrial dysfunction [25]. As an HDAC inhibitor [26], 4-PBA regulates glucose metabolism by reducing the enzymatic activity of HDAC5 [27], thereby modulating mitochondrial function through epigenetic mechanisms [28]. Further studies are needed to clarify the relationship between the direct HK2-targeting pathway identified in our study and the HDAC5-mediated epigenetic regulatory pathway reported in the literature.

Lactate, an important metabolic product produced by anaerobic respiration, except for participating in the energy metabolism, recent year’s study found it also participates in the regulation of many biological processes (BPs) via protein lactylation modification [12, 29]. Lactylation modification of proteins was first discovered by Zhao et al from the University of Chicago in 2019, which confirmed that lactate was not only a by-product of glycolysis but also a signaling molecule regulating multiple BPs [30]. Protein lactylation modification primarily occurred on lysine residues of proteins, using lactate as a substrate to add a lactyl group to the lysine residues of proteins. This modification may induce the structural changes of proteins, thereby influencing their functions and activities [31]. Yang *et al* found that in patients with hyperlactatemia, lactate activated the lysine acetyltransferase 8-phosphoenolpyruvate carboxykinase 2 (KAT8-PCK2) axes, leading to lactylation modification of PCK2. This modification exacerbated ferroptosis of hepatocytes induced by hepatic ischemia/reperfusion injury by reprograming Oxysterol-binding protein-related protein 2B (OXSM)-dependent mitochondrial fatty acid synthesis [32]. Zhou *et al.* discovered that in astrocytes, low-density lipoprotein receptor-related protein-1 (LRP1) promoted astrocyte-to-neuron mitochondrial transfer by decreasing lactate production and ADP-ribosylation factor 1 (ARF1) lactylation [33]. Our present study found that lactate levels were upregulated after sepsis, leading to lactylation modification of the cytoskeletal protein Arpc1b, which led to mitochondrial dynamic imbalance and mitochondrial dysfunction. 4-PBA restored mitochondrial function by decreasing lactate, and reducing in Arpc1b lactylation modification. In addition to Arpc1b lactylation, we also found that carbohydrate metabolic enzymes, endocytosis-related proteins, and other proteins in cardiomyocytes underwent lactylation modification after sepsis, but the specific mechanisms remain to be further investigated.

However, several limitations exist in this study: First, in terms of species differences, the study was conducted using rodent (mouse/rat) CLP models of sepsis. It still has obvious species limitations. There are significant differences between rodents and humans in immune response patterns, drug metabolism pathways, organ physiological structures after sepsis onset. Therefore, the clinical translational value of the research conclusions drawn only based on rodent models needs further verification. Second, the study focused on the acute therapeutic effect of 4-PBA within 12–24 h after surgery in septic model mice, focusing on observing its improvement effect on pathological damage in the acute phase of sepsis, but has not systematically explored the safety of its long-term application. The potential dose dependence (such as differences in safety under different administration doses) and potential adverse reactions of its long-term application have not been clarified. Therefore, in subsequent studies, we will focus on conducting long-term toxicity experiments of 4-PBA, systematically monitor the physiological indicators, organ function after long-term administration, clarify the safety boundary of its long-term application, and provide a reliable safety basis for subsequent clinical trials.

## Conclusions

In summary, our study showed that ERS inhibitor 4-PBA protected sepsis-induced cardiac dysfunction. The mechanism was related to 4-PBA inhibiting MAM formation, recovering mitochondrial dynamics balance and mitochondrial function. The mechanisms of PBA interacted with HK2 and then reduced the glycolysis and lactate production in myocardial cells which decreased the lactylation of cytoskeleton protein Arpc1b. The finding provides a new treatment measure and potential target for sepsis induced myocardial dysfunction.

## Supplementary Material

Supplementary_Figure_1_tkag039

Supplementary_Figure_2_tkag039

Supplementary_Figure_3_tkag039

Supplementary_Figure_4_tkag039

Supplementary_Figure_5_tkag039

Supplementary_Figure_6_tkag039

Supplementary_Figure_7_tkag039

Supplementary_Figure_8_tkag039

Supplementary_Figure_9_tkag039

Supplementary_Figure_10_tkag039

Supplementary_Figure_11_tkag039

Supplementary_Figure_12_tkag039

Supplementary_Figure_13_tkag039

Supplementary_Figure_14_tkag039

Supplemental_Tables_1-8_tkag039

Supplementary_material_tkag039

## Data Availability

All original data are available from the corresponding author upon request.
