# Supplementary material for: 4-phenylbutyric acid alleviated sepsis-induced myocardial dysfunction by inhibition of MAM formation to improve mitochondrial dynamic balance in myocardial cells
Source: Burns Trauma. 2026 Jun 3;14:tkag039. doi: 10.1093/burnst/tkag039 (PMC13526368; doi:10.1093/burnst/tkag039)
Supplement: Supplementary file 1 [file supplemental_tables_1-8_tkag039.docx]

**Supplemental Table 1. Plasma drug concentrations after a single administration in rats**

| Time(h) | plasma drug concentration (mg/L) | |
| --- | --- | --- |
|  | Mean | SD |
| 0 | 0 | 0 |
| 0.0833 | 14.11667 | 0.035119 |
| 0.25 | 13.78667 | 0.100664 |
| 0.5 | 13.30333 | 0.191398 |
| 1 | 12.39333 | 0.362261 |
| 2 | 10.75667 | 0.623244 |
| 4 | 8.116667 | 0.933506 |
| 8 | 4.65 | 1.051285 |
| 24 | 0.54 | 0.336006 |
| 48 | 0.025933 | 0.027 |

Plasma drug concentrations were measured at the indicated time points following a single dose administration in rats. Data are presented as mean ± SD (n=3).

**Supplemental Table 2. Pharmacokinetic parameters of the drug after a single administration in rats**

| parameters | unit | Mean | SD |
| --- | --- | --- | --- |
| AUC(0-t) | mg/L.h | 117.612 | 21.603 |
| AUC(0-∞) | mg/L.h | 117.824 | 21.834 |
| AUMC(0-t) | mg/L.h^2^ | 788.462 | 273.439 |
| AUMC(0-∞) | mg/L.h^2^ | 800.403 | 286.778 |
| MRT(0-t) | h | 6.567 | 1.12 |
| t1/2z | h | 4.997 | 1 |
| Tmax | h | 0.083 | 0 |
| CLz | L/h/kg | 0.043 | 0.008 |
| Cmax | mg/L | 14.117 | 0.035 |

Pharmacokinetic parameters were calculated from the plasma concentration-time data using non-compartmental analysis. Data are expressed as mean ± SD.

**Supplemental Table 3. Plasma drug concentrations after repeat administration in rats**

| Time(h) | plasma drug concentration (mg/L) | |
| --- | --- | --- |
|  | Mean | SD |
| 0 | 0 | 0 |
| 0.0833 | 17.46 | 0.067 |
| 0.25 | 17.059 | 0.063 |
| 0.5 | 16.475 | 0.059 |
| 1 | 15.367 | 0.051 |
| 2 | 13.368 | 0.038 |
| 4 | 10.117 | 0.023 |
| 8 | 5.794 | 0.019 |
| 24 | 0.624 | 0.009 |
| 48 | 0.022 | 0.001 |

Plasma drug concentrations were measured at the indicated time points following repeat dose administration in rats. Data are presented as mean ± SD (n=3).

**Supplemental Table 4. Pharmacokinetic parameters of the drug after repeat administration in rats**

| parameters | unit | Mean | SD |
| --- | --- | --- | --- |
| AUC(0-t) | mg/L.h | 145.528 | 0.454 |
| AUC(0-∞) | mg/L.h | 145.69 | 0.46 |
| AUMC(0-t) | mg/L.h^2^ | 956.385 | 6.822 |
| AUMC(0-∞) | mg/L.h^2^ | 964.341 | 7.265 |
| MRT(0-t) | h | 6.572 | 0.027 |
| t1/2z | h | 4.993 | 0.026 |
| Tmax | h | 0.083 | 0 |
| CLz | L/h/kg | 0.034 | 0 |
| Cmax | mg/L | 17.417 | 0.015 |

Pharmacokinetic parameters were calculated from the plasma concentration-time data using non-compartmental analysis. Data are expressed as mean ± SD.

**Supplemental Table 5. Plasma drug concentrations after a single administration in mice**

| Time(h) | plasma drug concentration（mg/L） | |
| --- | --- | --- |
|  | Mean | SD |
| 0 | 0 | 0 |
| 0.0833 | 17.647 | 0.045 |
| 0.25 | 17.233 | 0.126 |
| 0.5 | 16.633 | 0.241 |
| 1 | 15.493 | 0.448 |
| 2 | 13.443 | 0.774 |
| 4 | 10.147 | 1.164 |
| 8 | 5.817 | 1.312 |
| 24 | 0.68 | 0.421 |
| 48 | 0.032 | 0.034 |

Plasma drug concentrations were measured at the indicated time points following a single dose administration in mice. Data are presented as mean ± SD (n=3).

**Supplemental Table 6. Pharmacokinetic parameters of the drug after a single administration in mice**

| parameters | unit | Mean | SD |
| --- | --- | --- | --- |
| AUC(0-t) | mg/L.h | 147.158 | 26.985 |
| AUC(0-∞) | mg/L.h | 147.425 | 27.276 |
| AUMC(0-t) | mg/L.h^2^ | 988.306 | 342.17 |
| AUMC(0-∞) | mg/L.h^2^ | 1003.245 | 358.859 |
| MRT(0-t) | h | 6.579 | 1.119 |
| t1/2z | h | 5.004 | 1.002 |
| Tmax | h | 0.083 | 0 |
| CLz | L/h/kg | 0.035 | 0.007 |
| Cmax | mg/L | 17.647 | 0.045 |

Pharmacokinetic parameters were calculated from the plasma concentration-time data using non-compartmental analysis. Data are expressed as mean ± SD.

**Supplemental Table 7. Plasma drug concentrations after repeat administration in mice**

| Time(h) | plasma drug concentration（mg/L） | |
| --- | --- | --- |
|  | Mean | SD |
| 0 | 0 | 0 |
| 0.0833 | 23.37 | 1.883 |
| 0.25 | 23.057 | 2.145 |
| 0.5 | 22.11 | 2.054 |
| 1 | 20.947 | 2.136 |
| 2 | 17.573 | 1.725 |
| 4 | 13.197 | 1.026 |
| 8 | 8.563 | 1.305 |
| 24 | 0.85 | 0.246 |
| 48 | 0.041 | 0.025 |

Plasma drug concentrations were measured at the indicated time points following repeat dose administration in mice. Data are presented as mean ± SD (n=3).

**Supplemental Table 8. Pharmacokinetic parameters of the drug after repeat administration in mice**

| parameters | unit | Mean | SD |
| --- | --- | --- | --- |
| AUC(0-t) | mg/L.h | 201.789 | 25.61 |
| AUC(0-∞) | mg/L.h | 202.1 | 25.824 |
| AUMC(0-t) | mg/L.h^2^ | 1349.024 | 237.682 |
| AUMC(0-∞) | mg/L.h^2^ | 1366.571 | 249.988 |
| MRT(0-t) | h | 6.66 | 0.345 |
| t1/2z | h | 5.159 | 0.419 |
| Tmax | h | 0.083 | 0 |
| CLz | L/h/kg | 0.025 | 0.003 |
| Cmax | mg/L | 23.37 | 1.883 |

Pharmacokinetic parameters were calculated from the plasma concentration-time data using non-compartmental analysis. Data are expressed as mean ± SD.
