# Supplementary material for: 4-phenylbutyric acid alleviated sepsis-induced myocardial dysfunction by inhibition of MAM formation to improve mitochondrial dynamic balance in myocardial cells
Source: Burns Trauma. 2026 Jun 3;14:tkag039. doi: 10.1093/burnst/tkag039 (PMC13526368; doi:10.1093/burnst/tkag039)
Supplement: Supplementary file 2 [file supplementary_figure_10_tkag039.docx]

**Figure S10: Effects of skeletal proteins lactylation on MAMs in cardiomyocytes (H9C2).**

**(a)** Effects of mutant plasmids of Anxa6-K299, Arpc1b-K308, Cttn-K255, Cald1-K524, Septin5-K10 and Septin9-K154 on the formation of MAM in H9C2 cells (bar=20μm); **(b)** Statistics of the formation of MAM. Overlap coefficient=area of the formation of MAM /total area of the mitochondria and the endoplasmic reticulum(n=3).
