# Supplementary material for: 4-phenylbutyric acid alleviated sepsis-induced myocardial dysfunction by inhibition of MAM formation to improve mitochondrial dynamic balance in myocardial cells
Source: Burns Trauma. 2026 Jun 3;14:tkag039. doi: 10.1093/burnst/tkag039 (PMC13526368; doi:10.1093/burnst/tkag039)
Supplement: Supplementary file 3 [file supplementary_figure_11_tkag039.docx]

**Figure S11: Verification of lactylation modification of Arpc1b-K308 and mutants’ construction in H9C2 cells.**

**(a)** Statistics of the lactylation expression of Arpc1b-K308(n=3); **(b)** Expressions of Arpc1b-K308T and Arpc1b-K308R in H9C2 cells(n=3); **(c)** Fluorescence photograph of H9C2 cells 72 h and 2 weeks after lentiviral transfection (bar=40μm, n=3). **: p<0.01, compared with the control group; #: p<0.05, compared with the sepsis group.
