# Supplementary material for: 4-phenylbutyric acid alleviated sepsis-induced myocardial dysfunction by inhibition of MAM formation to improve mitochondrial dynamic balance in myocardial cells
Source: Burns Trauma. 2026 Jun 3;14:tkag039. doi: 10.1093/burnst/tkag039 (PMC13526368; doi:10.1093/burnst/tkag039)
Supplement: Supplementary file 4 [file supplementary_figure_12_tkag039.docx]

**Figure S12: Effects of Arpc1b-K308 lactylation on MAM and mitochondrial function in H9C2 cells.**

**(a)** Statistics of the formation of MAM. Overlap coefficient=area of the formation of MAM /total area of the mitochondria and the endoplasmic reticulum(n=3); **(b)** Effects of Arpc1b-K308 lactylation on ROS; **(c)** Effects of Arpc1b-K308 lactylation on mitochondrial membrance potential. **: p<0.01, compared with the control group; #: p<0.05, compared with the Sep ser group; @: p<0.05, compared with the Sep ser+PBA.
