# Supplementary material for: 4-phenylbutyric acid alleviated sepsis-induced myocardial dysfunction by inhibition of MAM formation to improve mitochondrial dynamic balance in myocardial cells
Source: Burns Trauma. 2026 Jun 3;14:tkag039. doi: 10.1093/burnst/tkag039 (PMC13526368; doi:10.1093/burnst/tkag039)
Supplement: Supplementary file 5 [file supplementary_figure_13_tkag039.docx]

**Figure S13: Effects of Arpc1b-K308 lactylation on heart function, mitochondrial dynamic balance and mitochondrial function in mice**.

**(a)** Validation of AAV9-mediated cardiac-specific targeting efficiency. Immunofluorescence staining of cardiac frozen sections from mice administered with AAV9-Arpc1b-EGFP via tail vein (scale bar=40 μm). **(b-c)** Effects of Arpc1b-K308 lactylation on mitochondrial structure in isolated cardiomyocytes. **(d-e)** Effects of Arpc1b-K308 lactylation on mitochondrial function in myocardial tissue. **(f)** Effect of Arpc1b-K308 lactylation on the pathology of mouse myocardial tissue (bar =100 μm)；**(g-h)** Effects of Arpc1b-K308 lactylation on cardiac contractile function. *: p<0.05; **:p<0.01; ns: no significant difference. Sham: Sham operation group; Sepsis: Sepsis group; Sepsis+PBA: Sepsis mice treated with 5 mg/kg 4-PBA infusion. Viruses: AAV9-CTNT-mArpc1b-3Flag-P2A-EGFP (Vector); AAV9-CTNT-mArpc1b(K308R)-3Flag-P2A-EGFP (K308R); AAV9-CTNT-mArpc1b(K308T)-3Flag-P2A-EGFP (K308T).
