# Supplementary material for: 4-phenylbutyric acid alleviated sepsis-induced myocardial dysfunction by inhibition of MAM formation to improve mitochondrial dynamic balance in myocardial cells
Source: Burns Trauma. 2026 Jun 3;14:tkag039. doi: 10.1093/burnst/tkag039 (PMC13526368; doi:10.1093/burnst/tkag039)
Supplement: Supplementary file 6 [file supplementary_figure_14_tkag039.docx]

**Figure S14: Proteomic analysis of cardiomyocytes (H9C2 cells).**

**(a)** Principal component analysis (PCA) plot; **(b)** Statistical chart of the significance of differences and fold changes in protein expression between the 4-PBA group and the sepsis group; **(c)** Pathways enriched by Gene Set Enrichment Analysis (GSEA); **(d)** GSEA enrichment analysis diagrams of the glycolysis/gluconeogenesis and pyruvate metabolism pathways respectively. The green curve represents the enrichment score; **(e)** Statistical chart of the expression of HK2 protein (n=3). **: p<0.01, compared with the control group; ##: p<0.01.
