# Supplementary material for: 4-phenylbutyric acid alleviated sepsis-induced myocardial dysfunction by inhibition of MAM formation to improve mitochondrial dynamic balance in myocardial cells
Source: Burns Trauma. 2026 Jun 3;14:tkag039. doi: 10.1093/burnst/tkag039 (PMC13526368; doi:10.1093/burnst/tkag039)
Supplement: Supplementary file 7 [file supplementary_figure_1_tkag039.docx]

**Figure S1: The transcriptome data of peripheral blood from septic patients.**

**(a)** Bioinformatic analysis workflow of the GSE65682 dataset. The bioinformatic pipeline for identifying differentially expressed genes (DEGs) in sepsis using the GSE65682 dataset, which includes peripheral blood samples from 42 healthy controls and 760 sepsis patients. After data processing and analysis, DEGs were identified and subjected to functional enrichment analysis to explore the biological processes underlying sepsis pathogenesis. **(b)** Principal component analysis (PCA) of septic patients and healthy individuals; **(c)** Volcano plot of differentially expressed genes; **(d-e)** Optimal thresholds; **(f)** Dendrogram based on gene dissimilarity; **(g)** Module-trait relationships in Weighted Gene Co-expression Network Analysis (WGCNA); **(h)** Scatter plots of the key modules; **(i)** Functional enrichment plot of the red module genes.
