# Supplementary material for: 4-phenylbutyric acid alleviated sepsis-induced myocardial dysfunction by inhibition of MAM formation to improve mitochondrial dynamic balance in myocardial cells
Source: Burns Trauma. 2026 Jun 3;14:tkag039. doi: 10.1093/burnst/tkag039 (PMC13526368; doi:10.1093/burnst/tkag039)
Supplement: Supplementary file 8 [file supplementary_figure_2_tkag039.docx]

**Figure S2: The transcriptome data of myocardial tissues from patients with septic cardiomyopathy.**

**(a)** Hierarchical clustering heatmap showing differentially expressed proteins of GSE79962; **(b)** GO enrichment analysis of different cell populations; **(c)** Box plot of the ERS scores of the sham and the sepsis group;**(d)** Box plot of the contraction scores in sham and sepsis; **(e)** tSNE plot of contractile and non-contractile cardiomyocytes; nonconCM: non-contractile cardiomyocytes; conCM: contractile cardiomyocytes. ERS score: endoplasmic reticulum stress score. contraction score: myocardial cell contraction score.
