# Supplementary material for: 4-phenylbutyric acid alleviated sepsis-induced myocardial dysfunction by inhibition of MAM formation to improve mitochondrial dynamic balance in myocardial cells
Source: Burns Trauma. 2026 Jun 3;14:tkag039. doi: 10.1093/burnst/tkag039 (PMC13526368; doi:10.1093/burnst/tkag039)
Supplement: Supplementary file 9 [file supplementary_figure_3_tkag039.docx]

**Figure S3: Dose–response analysis of 4-PBA in a rat model of sepsis-induced cardiomyopathy and pharmacokinetics in rats and mice.**

**(a–c)** Cardiac function parameters, including left ventricular ejection fraction (LVEF, a), left ventricular fractional shortening (LVFS, b), and cardiac output (CO, c), were measured at 12 after sepsis induction. 4-PBA treatment dose-dependently improved cardiac contractility, with significant effects observed at doses ≥ 1 mg/kg. **(d–f)** Serum levels of myocardial injury markers, cardiac troponin I (cTnI, d), aspartate aminotransferase (AST, e), and alanine aminotransferase (ALT, f), were reduced by 4-PBA in a dose-dependent manner. **(g, h)** Renal function parameters, blood urea nitrogen (BUN, g) and serum creatinine (Scr, h), were measured to assess systemic organ protection. 4-PBA treatment mitigated sepsis-induced renal dysfunction at higher doses. **(i)** Kaplan–Meier survival curves of mice subjected to sepsis. 4-PBA treatment significantly improved survival in a dose-dependent manner. Data are presented as mean ± SEM. **(j-m)** Plasma concentration-time curves of single and repeated administration in rats and mice. **(n)** Detection of TNF-α in H9C2 by ELISA(n=8). **(o)** Detection of IL-1β in H9C2 by ELISA(n=8). **:p<0.01, compared with the control group; #:p＜0.05, ##: p<0.01, compared with sepsis serum group.
