# Supplementary material for: 4-phenylbutyric acid alleviated sepsis-induced myocardial dysfunction by inhibition of MAM formation to improve mitochondrial dynamic balance in myocardial cells
Source: Burns Trauma. 2026 Jun 3;14:tkag039. doi: 10.1093/burnst/tkag039 (PMC13526368; doi:10.1093/burnst/tkag039)
Supplement: Supplementary file 10 [file supplementary_figure_4_tkag039.docx]

**Figure S4: Effects of** **4-PBA on the mitochondrial function of sepsis serum treated H9C2 cells.**

**(a)** Statistical chart on mitochondrial membrane potential(∆Ψm) (n=3); **(b)** Statistical chart on OCR (n=3); **(c)** Effect of 4-PBA on ATP concentration(n=8); **(d)** Effect of 4-PBA on ROS (bar=25μm, n=3). **:p<0.01, compared with the control group; #:p＜0.05, ##: p<0.01, compared with sepsis serum group. ROS: reactive oxygen species; OCR: mitochondrial oxygen consumption rate; Ctrl: Control group; Sep ser: Group stimulated by sepsis serum; Sep ser+PBA1: After stimulation with sepsis serum, 1 mmol/L of 4-PBA is added; Sep ser+PBA5: After stimulation with sepsis serum, 5 mmol/L of 4-PBA is added.
