# Supplementary material for: 4-phenylbutyric acid alleviated sepsis-induced myocardial dysfunction by inhibition of MAM formation to improve mitochondrial dynamic balance in myocardial cells
Source: Burns Trauma. 2026 Jun 3;14:tkag039. doi: 10.1093/burnst/tkag039 (PMC13526368; doi:10.1093/burnst/tkag039)
Supplement: Supplementary file 11 [file supplementary_figure_5_tkag039.docx]

**Figure S5: Effects of 4-PBA on the expression of mitochondrial dynamin proteins in sepsis serum treated cardiomyocytes (H9C2).**

**(a)** Detection of the expression of mitochondrial fission and fusion proteins by Western Blot (n=3); **(b-c)** Changes in the expression of mitochondrial fusion proteins Mfn2 and OPA1(n=3); **(d-e)** Effects of 4-PBA on mitochondrial DRP1 and cytoplasmic DRP1(n=3). **: p<0.01, compared with the sham operation group. Cyto DRP1: DRP1 in the cytoplasm; mito DRP1: DRP1 in the mitochondria. Ctrl: Control group; Sep ser: Group stimulated by sepsis serum; Sep ser+PBA: After stimulation with sepsis serum, 5 mmol/L of 4-PBA is added.
