# Supplementary material for: 4-phenylbutyric acid alleviated sepsis-induced myocardial dysfunction by inhibition of MAM formation to improve mitochondrial dynamic balance in myocardial cells
Source: Burns Trauma. 2026 Jun 3;14:tkag039. doi: 10.1093/burnst/tkag039 (PMC13526368; doi:10.1093/burnst/tkag039)
Supplement: Supplementary file 12 [file supplementary_figure_6_tkag039.docx]

**Figure S6: Metabolomics analysis of myocardial cells (H9C2) treated with sepsis serum and 4-PBA.**

**(a)** Principal component analysis (PCA) of different groups. Each scatter represents a sample. **(b)** Orthogonal partial least squares discriminant analysis (OPLS-DA).
