# Supplementary material for: 4-phenylbutyric acid alleviated sepsis-induced myocardial dysfunction by inhibition of MAM formation to improve mitochondrial dynamic balance in myocardial cells
Source: Burns Trauma. 2026 Jun 3;14:tkag039. doi: 10.1093/burnst/tkag039 (PMC13526368; doi:10.1093/burnst/tkag039)
Supplement: Supplementary file 13 [file supplementary_figure_7_tkag039.docx]

**Figure S7: Role of lactic acid in the regulation of MAM and mitochondrial function in cardiomyocytes (H9C2) treated with sepsis serum and 4-PBA.**

**(a)** The formation of MAM in H9C2 cells treated with sepsis serum, 4-PBA (5mM) and lactic acid (bar=20μm, n=3). The overlap coefficient=the formation of MAM/the total area of mitochondria and the endoplasmic reticulum. **(b)** Effect of lactate on mitochondrial membrane potential(∆Ψm) (bar=25μm, n=3); **(c)** Effect of lactate on ATP content(n=8); **(d)** Effect of lactate on OCR(n=3); **(e)** The effect of lactate on ROS (bar=25μm, n=3). **: p<0.01, compared with Ctrl group; #: p<0.05, ##: p<0.01, compared with the sepsis serum group; @: p<0.05, @@: p<0.01, compared with the 4-PBA treatment group. Ctrl: Control group; Sep ser: Group stimulated by sepsis serum; ROS: reactive oxygen species; OCR: mitochondrial oxygen consumption rate; ER-Mito: Contact between the endoplasmic reticulum and mitochondria; ER: Endoplasmic reticulum; Mito: Mitochondria. Sep ser+PBA: After stimulation with sepsis serum, 5 mmol/L of 4-PBA is added; Sep ser+PBA+lac: After stimulation with sepsis serum, 5 mmol/L of 4-PBA and 10Mm lactate are added.
