# Supplementary material for: 4-phenylbutyric acid alleviated sepsis-induced myocardial dysfunction by inhibition of MAM formation to improve mitochondrial dynamic balance in myocardial cells
Source: Burns Trauma. 2026 Jun 3;14:tkag039. doi: 10.1093/burnst/tkag039 (PMC13526368; doi:10.1093/burnst/tkag039)
Supplement: Supplementary file 14 [file supplementary_figure_8_tkag039.docx]

**Figure S8: Effects of knockout MCT1 on mitochondrial dynamics, mitochondrial function and heart function in septic mice.**

**(a)** Validation of MCT1 knockout efficiency; **(b)** Effects of MCT1 knockout on ATP content (n=8); **(c)** Effects of MCT1 knockout on ROS (n=8). **: p<0.01, compared with the control group; ##: p<0.01, compared with the sepsis group.
