# Supplementary material for: 4-phenylbutyric acid alleviated sepsis-induced myocardial dysfunction by inhibition of MAM formation to improve mitochondrial dynamic balance in myocardial cells
Source: Burns Trauma. 2026 Jun 3;14:tkag039. doi: 10.1093/burnst/tkag039 (PMC13526368; doi:10.1093/burnst/tkag039)
Supplement: Supplementary file 15 [file supplementary_figure_9_tkag039.docx]

**Figure S9:** **Detection of lactylation modification in H9C2 cells treated with sepsis serum and 4-PBA.**

**(a)** PCA analysis; **(b)** The number of lactylation sites identified in different proteins.
