# Supplementary material for: 4-phenylbutyric acid alleviated sepsis-induced myocardial dysfunction by inhibition of MAM formation to improve mitochondrial dynamic balance in myocardial cells
Source: Burns Trauma. 2026 Jun 3;14:tkag039. doi: 10.1093/burnst/tkag039 (PMC13526368; doi:10.1093/burnst/tkag039)
Supplement: Supplementary file 16 [file supplementary_material_tkag039.zip › Supplementary Figure 3.docx]

Supplementary Figure 3: Dose–response analysis of 4-PBA in a rat model of sepsis-induced cardiomyopathy and pharmacokinetics in rats and mice
